# Stapled peptides as a new technology to investigate protein–protein interactions in human platelets†
†Electronic supplementary information (ESI) available. See DOI: 10.1039/c8sc00284c


**DOI:** 10.1039/c8sc00284c

**Published:** 2018-04-25

**Authors:** Jessica Iegre, Niaz S. Ahmed, Josephine S. Gaynord, Yuteng Wu, Kara M. Herlihy, Yaw Sing Tan, Maria E. Lopes-Pires, Rupam Jha, Yu Heng Lau, Hannah F. Sore, Chandra Verma, Daniel H. O' Donovan, Nicholas Pugh, David R. Spring

**Affiliations:** a Department of Chemistry , University of Cambridge , CB2 1EW , UK . Email: spring@ch.cam.ac.uk; b Department of Biomedical and Forensic Sciences , Anglia Ruskin University , CB1 1PT , UK . Email: nicholas.pugh@anglia.ac.uk; c Discovery Sciences , IMED Biotech Unit , AstraZeneca , Cambridge , UK; d Bioinformatics Institute , Agency for Science, Technology and Research (A*STAR) , 30 Biopolis Street, #07-01 Matrix , 13867 , Singapore; e School of Chemistry , The University of Sydney , NSW 2006 , Australia; f Department of Biological Sciences , National University of Singapore , 14 Science Drive 4 , Singapore 117543; g School of Biological Sciences , Nanyang Technological University , 60 Nanyang Drive , Singapore 637551; h Oncology, IMED Biotech Unit , AstraZeneca , Cambridge , UK

## Abstract

Platelets are blood cells with numerous crucial pathophysiological roles in hemostasis, cardiovascular thrombotic events and cancer metastasis. Platelet activation requires the engagement of intracellular signalling pathways that involve protein–protein interactions (PPIs). A better understanding of these pathways is therefore crucial for the development of selective anti-platelet drugs. New strategies for studying PPIs in human platelets are required to overcome limitations associated with conventional platelet research methods. For example, small molecule inhibitors can lack selectivity and are often difficult to design and synthesise. Additionally, development of transgenic animal models is costly and time-consuming and conventional recombinant techniques are ineffective due to the lack of a nucleus in platelets. Herein, we describe the generation of a library of novel, functionalised stapled peptides and their first application in the investigation of platelet PPIs. Moreover, the use of platelet-permeable stapled Bim BH3 peptides confirms the part of Bim in phosphatidyl-serine (PS) exposure and reveals a role for the Bim protein in platelet activatory processes. Our work demonstrates that functionalised stapled peptides are a complementary alternative to conventional platelet research methods, and could make a significant contribution to the understanding of platelet signalling pathways and hence to the development of anti-platelet drugs.

## Abstract 2

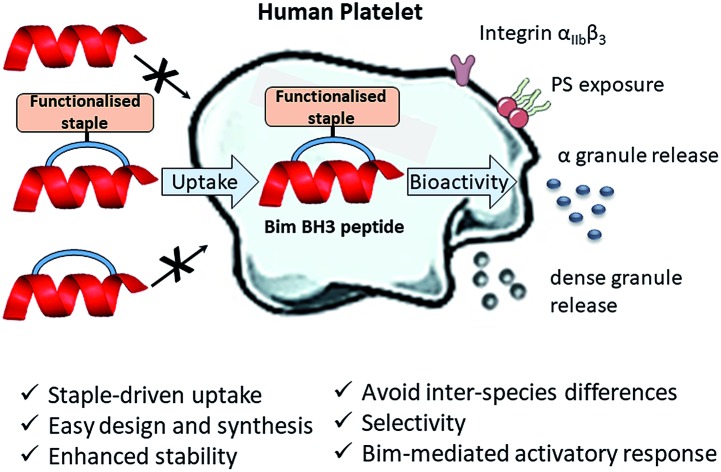
We describe the first application of stapled peptides in human platelets. Bim BH3 stapled peptides are used to overcome the limitations of traditional methods and uncover a new role for Bim in platelet activation.

## Introduction

Platelets are blood cells that are maintained in the circulation in an inactive state, but initiate thrombus formation upon activation. Signalling processes during platelet activation consist of complex and finely regulated protein–protein interaction (PPI) networks which have been the subject of considerable research in recent years.^1^,^2^ The understanding of PPIs in platelets may assist in the discovery of selective and specific anti-platelet therapeutics which find application in the treatment of cardiovascular diseases and cancer therapy. However, current methodologies used to investigate PPIs in human platelets have several limitations. As platelets are anuclear, conventional recombinant techniques to study the roles of proteins in platelets are ineffective. Other experimental approaches include the use of small molecule inhibitors and the generation and use of transgenic animals.^3^ The use of small molecules is disadvantaged by the potential for off-target effects, derived from the lack of specificity among related targets.^4^ Moreover, the design and development of small molecules targeting a shallow protein–protein interface can be remarkably challenging despite some recent advances.^5^ Conversely, generating transgenic animal models is time-consuming and costly, may be prone to side-effects during embryonic development, and may not accurately represent the human situation.^6^ It is clear that there is a need for new, complementary approaches that can be used in human platelets directly, are easily designed, relatively cheap, and selective.

Peptide-based therapeutics offer a viable solution since they are known to be potent and selective against biological targets that are otherwise difficult to manipulate with small molecules.^4^ Such approaches have been used to manipulate intracellular PPIs, wherein large and shallow contact areas of a protein are successfully targeted by peptides, which are able to mimic the native counter protein.^4^,^7^–^10^ Although highly potent and selective, peptides often suffer from poor cell permeability, plasma stability and hence poor bioavailability. To overcome the poor pharmacokinetic properties of linear peptides, stapled peptides have been successfully developed and used to target intracellular PPIs.^11^–^15^ Indeed, an all-hydrocarbon stapled peptide, ALRN-6924, is under clinical development as an anti-cancer drug targeting HDM2/p53.^16^ Among the techniques used for peptide stapling,^17^ the two-component double Cu-catalysed azide–alkyne cycloaddition (CuAAC) strategy constrains the peptides in the bioactive conformation and simultaneously improves pharmacokinetic properties. Moreover, this strategy uses unnatural azido amino acids that can be easily synthesised^18^ and facilitates the functionalisation of the staple with cell-permeabilising motifs, fluorescent-labelled tags and photo-switchable linkers.^19^ The independent functionalisation of the staple can be particularly useful when dealing with long and synthetically challenging peptide sequences, as the complex functionality is added to the staple rather than the N- or C-terminus of the peptide. In addition, this approach only requires one linear peptide to generate a variety of functionalised stapled peptides, facilitating the exploration of various functionalities on the linker and thus properties of the overall peptide.

Of particular interest in platelets is the investigation of PPIs of the BCL-2 family members, in particular the BH3-only protein family which has a key role in regulating intrinsic apoptosis and the lifetime of platelets.^20^–^23^ Whilst all-hydrocarbon stapled peptides corresponding to the BH3 domains of the BH3-only protein family members have previously been used to investigate apoptotic processes in haematological cancer cells, the efficacy of these molecules for use in platelets has yet to be investigated. The BH3-only family of proteins has been extensively studied and is therefore an ideal system to use to validate our stapled peptide technology in human platelets.

**Fig. 1:**
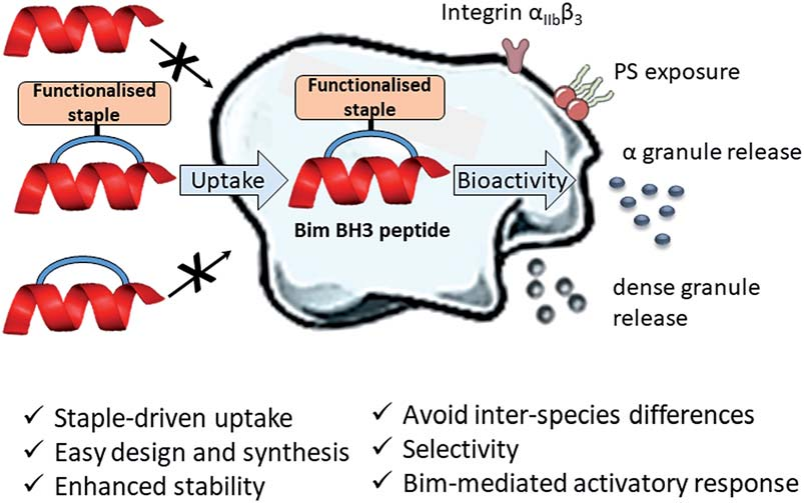
First application of functionalised stapled peptides in the investigation of signalling pathways in human platelets. Peptide (red) uptake is driven by the functionalised staple (orange). Once in the cytosol the peptide is able to exert its biological activity by disrupting platelet PPIs.

Herein, we describe the first application of stapled peptides in human platelets. We investigate the potential of functionalised stapled peptides to enter the platelet cytosol and disrupt biologically important intracellular processes involving BCL-2 proteins. We present a range of functionalised staples that promote peptide uptake and reveal a new role for Bim BH3 in platelet activation (Fig. 1 and discussed later). Our work aims to provide a complementary alternative to small molecule inhibitors and transgenic animal models in the investigation of intracellular signalling pathways in human platelets.

Furthermore, our research aims to act as the basis for the future development of anti-platelet peptide-therapeutics that have improved selectivity, enhanced stability, are easy to design, synthesise and functionalise and most importantly can be applied to human platelets directly.

## Results and discussion

### Platelet uptake of the model peptides

**Fig. 2:**
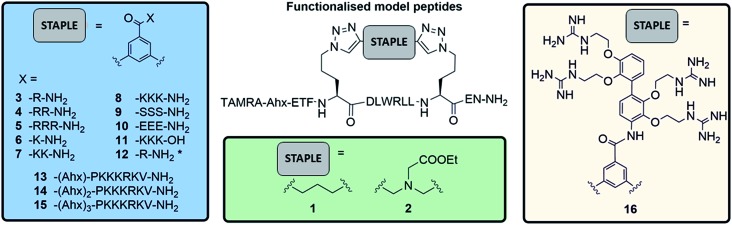
TAMRA labelled functionalised stapled peptides used to analyse platelet uptake of peptides. Schematics of the different peptidic (blue), non-peptidic functionalised staples (green) and a novel SMoC staple (pink). * indicates unlabelled peptides (N-terminus capped as acetyl).

To investigate the potential of functionalised staples to modulate the uptake of stapled peptides into platelets, we used a panel of fifteen TAMRA-labelled model peptides stapled with functionalised peptidic and non-peptidic groups (Fig. 2). The model peptides used are based on the p53 sequence (a nuclear effector) and should therefore have no effect on anucleated platelets. Functionalisation of the peptides was easily achieved using two-component CuAAC stapling technique, which allowed incorporation of functionalised groups into the dialkynyl linker, rather than to the N- or C-terminus of the peptide sequence.^24^ This approach makes functionalisation of the peptides easier since it requires the synthesis of one linear peptide, with the functionality being provided by the staple. Functionalised groups included: polycationic peptidic chains,^17^ the nuclear localisation sequences PKKRKV (NLS) derived from SV40 large T-antigen and a novel polyguanidine small molecule carrier (SMoC).^25^–^30^ Additionally, a panel of novel anionic and polar peptidic chains was also screened (Fig. 2).

**Fig. 3:**
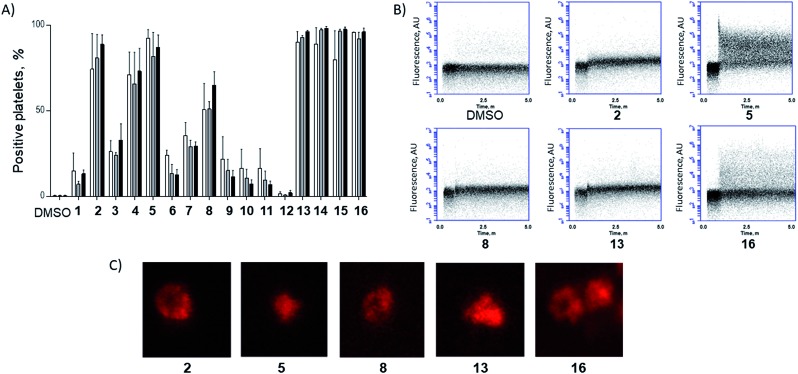
A functionalised staple is required to induce peptide uptake into platelets. (A) Platelets were incubated with TAMRA labelled model peptides (10 μM final concentration) for 1 h (white), 2 h (grey) and 3 h incubation (black) and the percentage positive for TAMRA fluorescence was quantified using flow cytometry. (B) Peptides showing high uptake within the first hour were analysed during first 5 min following treatment. Changes in platelet-associated fluorescence were recorded in real time using flow cytometry. Unlabelled platelets were recorded for 30 s prior to addition of the given peptide. (C) Platelets were incubated with 10 μM of TAMRA-model peptides for 1 h prior to imaging by confocal microscopy.‡

Washed platelet suspensions were incubated with peptides for varying periods of time and cellular uptake was quantified using flow cytometry. Treatment with **13–15** (carrying NLS) or **16** (bearing the SMoC motif) showed the highest platelet-associated fluorescence after a 3 hour incubation (96.1 ± 0.8%, 98.1 ± 1.1%, 97.5 ± 1.4% and 96.1 ± 2.2% positive platelets for peptides **13** to **16** respectively), indicating significant association of the platelets with the stapled peptide. Peptide **2** (ethyl ester) and the poly-arginine stapled peptides **4** and **5** showed intermediate uptake after 3 hours (88.8 ± 5.6%, 73.0 ± 13.3% and 87.0 ± 7.3% respectively for **2**, **4** and **5**). Only 34.9 ± 7.8% of platelets were fluorescent following **8** (poly-lysine) treatments after 3 hours. Changes in fluorescence were not observed with other peptides (Fig. 3A).

In order to explore the rate of peptide uptake, changes in platelet fluorescence were monitored during the first 5 minutes (Fig. 3B) and after 15, 30, 45 and 60 minutes following treatment with peptides (Fig. SI_1, ESI†). More than 50% of platelets became fluorescent during the first 15 minutes of incubation with peptides **13–16**, with a further increase after the first 45 minutes. Some 40–50% of platelets were fluorescent after 15 minutes following treatment with peptides **2**, **5** or **8**. No further increase was observed thereafter (Fig. SI_1, ESI†). Continuous assessment of peptide uptake in the first 5 minutes of treatment demonstrated that peptides **5** and **16** caused a rapid increase in platelet fluorescence (Fig. 3B).

Confocal imaging demonstrated that peptides **5** and **13** permeated into the platelet cytosol, whilst treatment with **2**, **8** or **16** showed predominant localisation to the platelet membrane (Fig. 3C).‡Platelets are considerably smaller than nucleated cells and therefore higher resolution in confocal microscopy cannot be achieved. Peptide uptake experiments of the most permeable model peptides **5**, **13–16** were repeated in the presence of platelet-rich plasma (PRP) to ensure realistic conditions (ESI, Fig. SI_2a†). The results showed only a small reduction in peptide uptake between 10 and 20% indicating that the uptake is not prevented by interaction with the other components of the plasma.

Conventional platelet activation responses were assessed to investigate potential off-target effects of the peptide treatment. Pleasingly, only low activation changes were observed following treatment with NLS-bearing peptides **13–15** and SMoC peptide **16** (Fig. SI_3, ESI†). No change in platelet activation markers was observed for the other peptides, indicating that the treatment of platelets with the model peptides did not result in non-specific responses. Significantly, undesired platelet aggregation was only observed in response to treatment with peptide **16**, but not for the other peptides (Fig. SI_4, ESI†). Thus, peptide **16** was excluded from further studies.

This data set provides evidence that functionalisation of stapled peptides is required for the uptake of peptides into platelets, with the NLS sequence (**13–15**) and poly-arginine staple (**5**) being the most effective. Further experiments were performed to investigate whether stapled peptides based on a biologically relevant sequence were able to elicit a relevant intracellular response in platelets.

### Effect of Bim BH3 peptides on platelet PS exposure and activation

Further experiments using peptides based around the Bim BH3 sequence were performed to determine whether platelet-permeable peptides retained bio-functionality. The role of BH3-only proteins in the development of a pro-coagulant platelet phenotype has been investigated using the Bad BH3 mimetic small molecule ABT737. This induces mitochondrial membrane depolarization, activation of caspases –9, –8 and –3, phosphatidyl serine (PS) exposure and inhibition of the platelet activation process by interacting indirectly with the pro-apoptotic protein Bax *via* inhibition of the pro-survival BCL-2 proteins.^31^–^34^ Previous work has demonstrated that a Bim sequence-based peptide (denoted Bim SAHBa) is able to induce Bax mediated apoptotic responses in nucleated cells.^35^–^37^ To the best of our knowledge, the implication of the interaction of Bim BH3 with antiapoptotic and proapoptotic Bcl-2 proteins in platelets is unknown, hence our interest in this PPI.

In order to demonstrate the efficacy of functionalised group-mediated peptide delivery in platelets, we synthesised two-component stapled peptides based on the SAHBa sequence and investigated the effects of these on platelet processes.

**Fig. 4:**
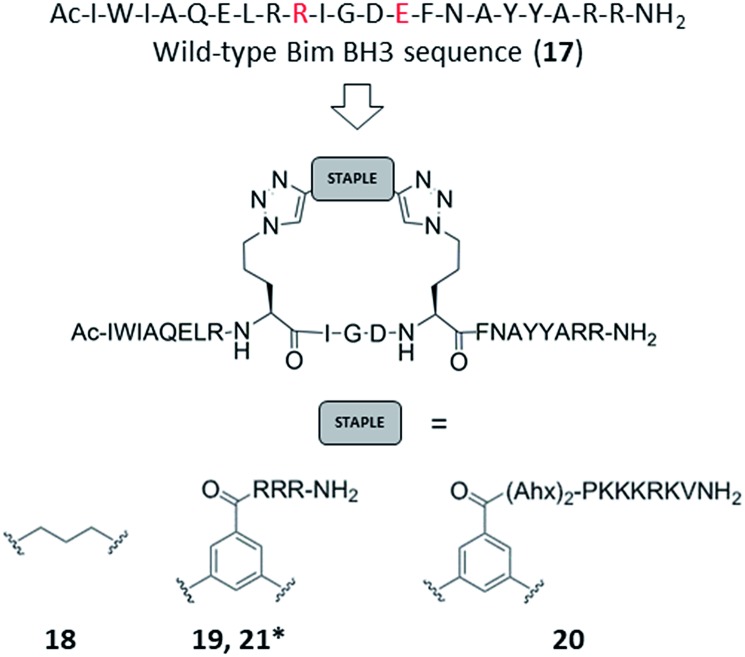
Bim BH3 stapled peptides. Peptide **17**: wild-type Bim BH3 sequence. Amino acids that have been replaced with azido amino acids to perform the CuAAC reactions are highlighted in red. * based on the R153D mutant with sequence Ac-I-W-I-A-Q-E-L-D-Orn(N_3_)-I-G-D-Orn(N_3_)-F-N-A-Y-Y-A-R-R-NH_2_.

**Fig. 5:**
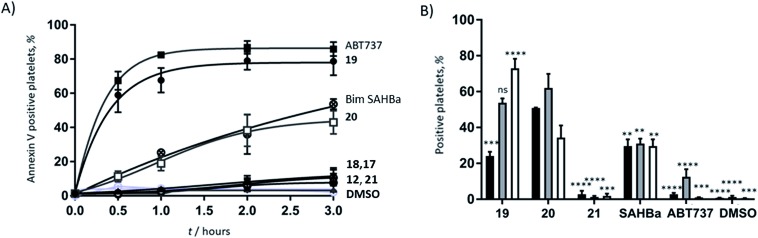
(A) Functionalised, stapled Bim BH3 peptides induce PS exposure in platelets. Platelets were treated with peptides and changes in annexin-V binding were quantified using flow cytometry. 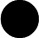 peptide **19**, 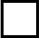 peptide **20**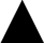 peptide **18**, 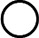 peptide **17**, 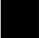 ABT737, 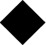 peptide **12**, 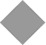 vehicle (DMSO), 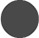 peptide **21**, 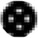 Bim SAHBa. (B) Stapled Bim BH3 peptides, but not ABT737 nor **21**, induce platelet activation. Platelet activation markers (% positive platelets) were investigated following 1 h of peptide treatment. 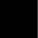 PAC-1, 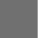 CD62P and 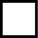 CD63 binding. Statistical significance compared to **20**: ****P < 0.0001, ***P between 0.0001 and 0.0005, **P between 0.0010 and 0.0050 ns = non-significant.

Azido ornithine was employed as the unnatural amino acid to perform the two-component double CuAAC stapling. This facilitated the synthesis of different stapled peptides from one linear precursor: peptide **18** with a non-functionalised staple, **19** with a poly-arginine staple and **20** with a NLS motif (Fig. 4). Peptides **19** and **20** contain the same functionalised staples as the model peptides with the highest cytosolic platelet uptake. The influence of these peptides on activatory processes in washed platelet suspensions was compared with ABT737, which causes PS exposure but does not activate platelets.^31^,^33^,^38^ Treatment of platelets with **19** or **20**, resulted in PS exposure (with 78 ± 1.3% and 42.9 ± 6.0% annexin V binding respectively after 3 hours, Fig. 5A), consistent with the generation of a pro-coagulant phenotype. Of note, **19** showed a similar profile to ABT737, the previously reported clinical candidate and appeared to be more potent than the all-hydrocarbon Bim peptide (Fig. 5A). No annexin-V binding was observed following treatment with the unstapled wild-type peptide **17** nor with the non-functionalised stapled peptide **18**. This can be explained by the poor membrane permeability of peptides **17** and **18** and is consistent with the behaviour of the non-functionalised model peptide **1** (Fig. 2, ESI Fig. SI_2b, SI_5†). Even though FITC-labelled peptide **18** showed high platelet fluorescence in plasma, confocal imaging showed the peptide forming aggregates outside the platelet cytosol and hence being unable to reach the target protein (ESI, Fig. SI_5†). Conversely, peptides **19** and **20** localized into the platelet cytosol with peptide **19** being the most effective (ESI, Fig. SI_5†). Mutation of arginine 153 to aspartic acid (R153D) in the Bim BH3 domain (IWIAQELDRIGDEFNAYYARR) is unable to cause PS exposure in nucleated cells.^35^ Therefore, we tested a R153D Bim stapled peptide functionalised with poly-R_3_ (**21**). This peptide did not result in PS exposure in platelets despite being platelet-permeable (Fig. 5A, ESI Fig. SI_2b, SI_5†), suggesting that the effects of the BH3 stapled peptide are specific to biologically relevant processes, and not due to non-specific, off target effects.

Circular dichroism experiments showed the biologically active stapled peptides to be helical, however there was no enhancement in helicity for the stapled peptide compared to the wild-type (Fig. SI_6, ESI†). Consistent with previous reports,^39^ peptides retained biological activity despite the lack of enhanced helicity in solution. Moreover, our results confirm that a permeability-enhancing staple is required for the peptides to reach the platelet cytosol, to effect bioactivity and to enhance stability in human serum (Fig. SI_7, ESI†). To investigate whether the stapling affects the ability of the peptides to bind to their target proteins, we ran molecular dynamics (MD) of an unfunctionalised form of peptides **19** and **20** in complex with an antiapoptotic BCL-2 protein which is known to be expressed in platelets, Bcl-xL.^20^ The simulations suggested that the stapled peptide retains an α-helical conformation upon binding and that its binding affinity is comparable to that of the unstapled wild-type peptide **17** (ESI Table SI_3, Fig. SI_10, SI_11, ESI†). Encouraged by these results, we measured the binding interaction of the wild-type and the stapled peptides in a SPR assay. The results confirm that the wild-type and platelet-permeable stapled peptide **19** are able to engage with Bcl-xL protein (relative *K*_Dapp_ 7.3 and 26 nM respectively, ESI, Table S1, Fig. SI_9†). Interestingly, the all-hydrocarbon Bim BH3 stapled peptide SAHBa showed a reduced binding affinity compared to the wild-type and peptide **19**.

As previous work has indicated that the small molecule ABT737 inhibits platelet activation, the effect of stapled peptides **19** and **20** on platelet activation markers was assessed.^40^–^42^ Both peptides induced integrin α_IIb_β_3_ activation (as measured by PAC-1 binding, Fig. 5B), whilst ABT737 was ineffective. Peptide **19** or **20** treatment resulted in 24.1 ± 2.4% and 50.8 ± 0.3% PAC1-positive platelets, respectively after 1 hour. Treatment with either peptides resulted in similar levels of CD62P expression (53.7 ± 2.5% and 62.1 ± 7.8%, for **19** and **20** respectively), indicating peptide-induced α granule secretion. Additionally, both peptides increased CD63 expression (correlated with dense granule release), with peptide **19** being more effective (72.9 ± 5.3% compared to 34.4 ± 6.7% for peptide **20**). Pleasingly, nonspecific activation was not observed when the mutant R15D (**21**) was used. Despite having similar effects on PS exposure, no increase in any activation marker was observed following treatment with ABT737. Whilst all the mimetics are able to induce PS exposure, only the peptides induce upregulation of platelet activation markers. Therefore, this data highlights that the mechanistic differences between the Bad BH3 mimetic ABT737 and the Bim BH3 mimetic stapled peptides **19** and **20** have different effects on platelet activatory responses and therefore might find different therapeutic applications.

## Conclusions

This work is the first to describe the use of functionalised stapled peptides to perturb and investigate signalling pathways in human platelets. We demonstrate the benefits of using the two-component CuAAC stapling technique to access a variety of novel functionalised peptides from a synthesised linear sequence. The nature of the motif (which is easily incorporated onto the staple) impacts the ability of the peptides to enter the platelet cytosol and modulate activity. This technique is particularly powerful when long and complex sequences are synthesised by solid-phase peptide synthesis as the cell-permeable motif can be added onto the staple and therefore, does not require the introduction of further complexity on the linear sequence. We synthesised Bim BH3 mimetic peptides to investigate PS exposure in platelets. The bioactivity of **19** was comparable to that of the small molecule Bad mimetic ABT737.

Importantly, unlike ABT737, stapled peptides **19** and **20** (both Bim mimetics) caused platelet activation, demonstrating differential activation of signalling pathways. Further studies are currently underway in our labs to elucidate these mechanistic differences. Perturbation of the BCL-2 pathway demonstrates the considerable potential of this technology to investigate other PPIs in human platelets and for future development of new anti-platelet drugs. One of the advantages of this technology over small molecule use is the ease of design and synthesis of the peptides, as they are a mimetic of the natural components. Moreover, limitations such as the lack of membrane permeability and poor serum stability are overcome by the presence of the staple. The double click approach only requires one linear peptide to enable the generation of a variety of functionalised stapled peptides, which facilitates the exploration of various functionalities on the linker and thus the properties of the overall peptide. In addition, the easy functionalisation of the staple provides the opportunity to incorporate a variety of motifs that could be useful for biological testing (for example, fluorophores, biotin and sequences regulating controlled release) or for achieving selectivity. Most importantly, this methodology allows testing in human platelets directly, avoiding the inter-species difference, cost and time limitations of transgenic animal models.

In theory, it is feasible that any PPI could be disrupted and studied using functionalised peptides. Thus, the number of potential targets that are accessible to study are increased. In addition, considering the specificity and versatility of stapled peptides, and the importance of platelets in many pathological diseases,^43^,^44^ this work sets the basis for the future development of specific anti-platelet peptide therapeutics.

## Conflicts of interest

There are no conflicts to declare.

## Supplementary Material

Supplementary information
