# Supplementary material for: Stapled peptides as a new technology to investigate protein–protein interactions in human platelets
Source: Chem Sci. 2018 Apr 25;9(20):4638–43. doi: 10.1039/c8sc00284c (PMC5969508; doi:10.1039/c8sc00284c)
Supplement: Supplementary file 1 [file SC-009-C8SC00284C-s001.pdf]

Electronic Supplementary Material (ESI) for Chemical Science.
This journal is © The Royal Society of Chemistry 2018

Stapled peptides as a new technology to investigate protein-protein interactions in human platelets

Jessica Iegre, Niaz S. Ahmed, Josephine S. Gaynord, Yuteng Wu, Kara M. Herlihy, Yaw Sing Tan, Maria E. Lopes-Pires, Rupam Jha, Yu Heng Lau, Hannah F. Sore, Chandra Verma, Daniel H. O'Donovan, Nicholas Pugh* and David R. Spring*

Table of Contents

1. **Methods** 3
1.1 Preparation of washed platelet suspension 3
1.2 Flow cytometry 3
1.2.1 Uptake experiments in washed platelet suspension 3
1.2.2 Uptake experiments in platelet-rich plasma (PRP) 4
1.2.3 PS exposure and activation markers 5
1.3 Light transmission aggregometry 6
1.4 Confocal microscopy 6
1.5 Data analysis and statistics 7
1.6 Circular dichroism 7
1.7 Human serum stability test 8
1.8 Protein expression and purification 9
1.9 Surface Plasmon Resonance (SPR) 10
1.10 General chemistry experimental procedures 12
1.11 Synthesis of linear peptides 14
1.12 Synthesis of azido amino acids 14
1.13 Synthesis of peptidic staples 16
1.14 Synthesis of poly-guanidine biphenyl SMoC staple 17
1.15 Two-component double CuAAC peptide stapling 19
2. **Peptide mass-spectrometry characterization data** 20
3. **^{1}H and ^{13}C NMR spectra** 22
4. **Molecular modelling** 28
4.1 Preparation of structures 28
4.2 Molecular Dynamics 28
4.3 Binding free energy calculation 30

1. Methods

Ethical approval for this study was obtained from the Faculty Research Ethics Committee at Anglia Ruskin University, Cambridge, UK.

1.1 Preparation of washed platelet suspension

Human blood was collected from healthy volunteers, who had not taken medication for two weeks, following informed consent in accordance with the Declaration of Helsinki. Blood was collected into 11 mM sodium citrate and washed platelets were prepared as follows: Platelet-rich plasma (PRP) was obtained by centrifugation (240 g, 15 min). Prostaglandin E1 (PGE_1, 2 µM) was added to prevent premature platelet activation, and the plasma was centrifuged at 640 g for 15 min. Platelets were suspended in calcium free Tyrode‘s buffer (CFT buffer, containing: 140 mM NaCl, 5 mM KCl, 10 mM N-2-hydroxyethylpiperazine-N‘-2-ethanesulfonic acid (HEPES), 5 mM Glucose, 0.42 mM NaH_2PO_4, 12 mM $NaHCO_3$, titrated to pH 7.4 with NaOH) to a final concentration of $2x10^8$ platelet/mL and rested at 37 °C for 1 h prior to experimentation.

1.2 Flow cytometry

Treated washed platelet suspensions ($1x10^7$/mL) were analysed by flow cytometry using a C6 Accuri Flow Cytometer (Beckton Dickinson, UK), in the presence or absence of Annexin-V-PE (BD Biosciences, UK), anti-PAC-1 (BD Biosciences), anti-CD62 (BD Pharmingen, UK), or anti-CD-63P (BD Pharmingen). Peptides were used to a final concentration of 10 µM (1 mM DMSO stock solutions). At least 10,000 events were acquired per experiment. The entire platelet population was primarily gated by FSC.

1.2.1 Peptide uptake experiments in a washed platelet suspension

The washed platelet suspension was rested at 37 °C for 1 h before commencing the experiment. 250 µL of washed platelet suspension was incubated with 2.5 µL of fluorescently labelled peptide (1 mM DMSO stock). The suspension was kept in the dark at 37 °C and measurments taken at the designed time points (1 h, 2 h, 3 h). For real time measurments, the peptides were added to the 250 µL of washed platelet suspension after events were recorded for 1 minute and monitored for a further 4 min.

Flow cytometry was used to investigate model-peptide uptake over 60 minutes (Figure SI_1)

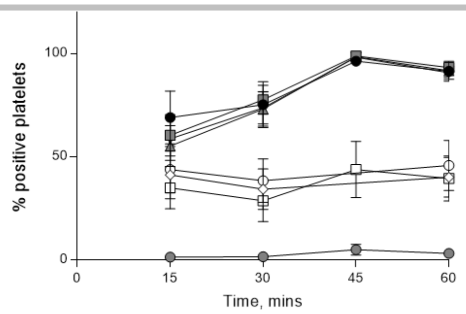

Figure SI_1. Peptide uptake of model peptides monitored during the first 60 minutes of treatment. ●: vehicle (DMSO), ●: **16,** ■: **15,** ▲: **14,** ▼: **13,** ◇: **8,** □: **5,** ○: **2**

1.2.2 Peptide uptake experiment in platelet rich plasma (PRP)

Human blood was collected from healthy volunteers, who had not taken medication for two weeks, following informed consent in accordance with the Declaration of Helsinki. Blood was collected into 11 mM sodium citrate and platelet rich plasma (PRP) was obtained by centrifugation (240 g, 15 min). Prostaglandin E1 (PGE_1, 2 µM) was added to prevent premature platelet activation. The PRP was rested for 30 minutes prior experimentation. 120 µL of PRP was added to 1.2 µL of fluorescently labelled peptide (1 mM DMSO stock) and incubated in the dark at 37 °C for 1 h and analysed by flow cytometry.

A comparison of peptide uptake of model peptides **5**, **13**-**16** after 1 h incubation in washed platelet suspension and PRP is shown in Figure SI_1a.

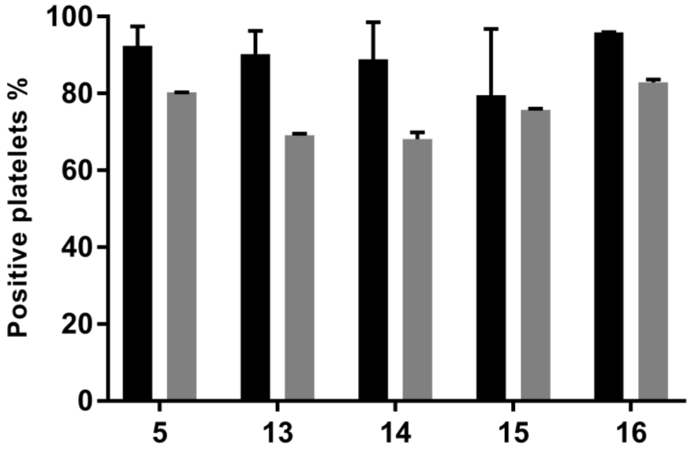

Figure SI_2a. Uptake of platelet-permeable model peptides **5**, **13**-**16** in washed platelet suspension (black) and PRP (grey). Labelled peptides were incubated to a final concentration of 10 µM for 1 h prior experimentation. The results are the avarage of three indepentent repeats and error shown as SEM.

Peptide uptake of FITC-labelled BH3 peptides **17**-**21** and SAHBa in PRP after 15, 30, 60 minutes of incubation is shown in Figure SI_2b.

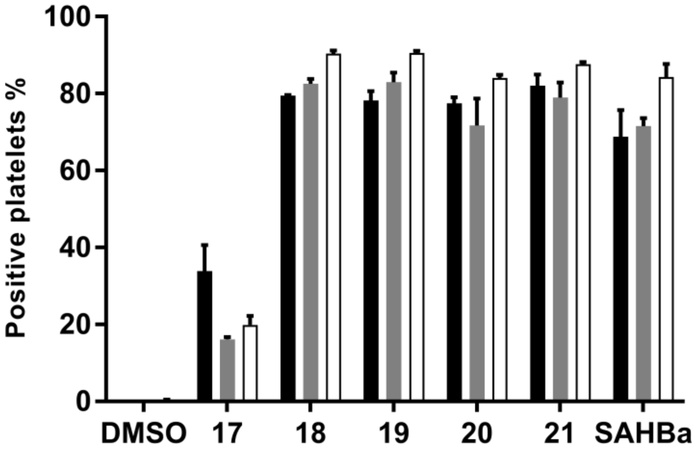

Figure SI_2b. Uptake of Bim BH3 peptides **17**-**21** and SAHBa in PRP after 15 minutes (black), 30 (grey), 1 h (white). Labelled peptides were incubated in plasma to a final concentration of 10 µM. The results shown are the avarage of two independent repeats and error shown as SEM.

1.2.3 PS exposure and activation markers

PS exposure was assessed after incubation of 50 µL of washed platelet suspensions at 37 °C in 445 µL of Tyrode's solution (pH 7.4) with the peptide (5 µL of 1 mM stock solution in DMSO). At the designed time point 25 µL of the suspension was added to 25 µL of Annexin V (10% Annexin V in Tyrode's solution) under the protection from direct light and analysed by flow cytometry.

Platelet activation markers (PAC-1, CD62P and CD63 binding) were assessed after incubation of 3 µL of of washed platelet suspensions at 37 °C in 105 µL of CFT buffer (pH 7.4) with 1.2 µL of the desired antibody and the peptide (1.1 µL of 1 mM stock solution in DMSO). The suspensions were analysed by flow cytometry.

Flow cytometry was used to analyse activation markers after treatment with the platelet permeable model peptides (Figure SI_3).

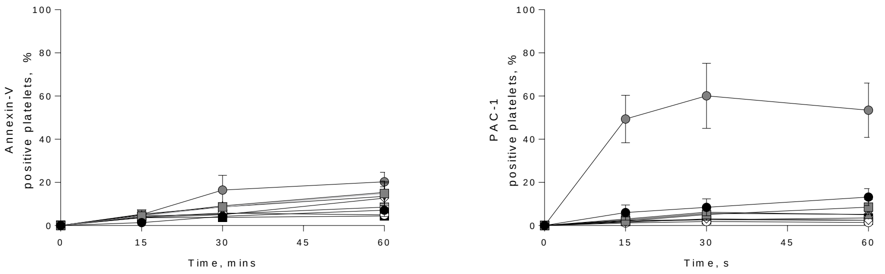

Figure SI_3. Left: Annexin V binding for model platelet-permeable peptides; Right: PAC-1 binding for model platelet-permeable peptides. ●: Thrombin 1U/mL, ■ vehicle (DMSO), ●: **16,** ■: **15,** ▲: **14,** ▼: **13,** ◇: **8,** □: **5,** ○: **2**

1.3 Light Transmission Aggregometry

Peptides were used to a final concentration of 10 µM (2.5 µL of 100 uM stock solution in DMSO). Platelet aggregation was monitored using an AggRam aggregometer (Helena Biosciences, UK). 250 µL of washed platelet suspensions ($1x10^8$/mL) were stimulated under stirring conditions at 37 °C for 15 mins and the percentage aggregation was acquired from the aggregation traces using proprietary software (Helena Biosciences, UK).

Aggregation results of platelets treated with cell-permeable model peptides are reported below.

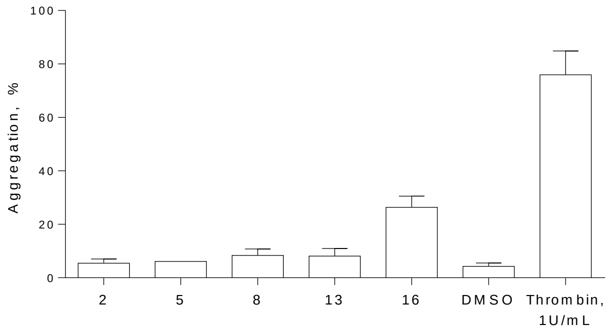

Figure SI_4; Platelet aggregatory responses to treatment with model peptides. Vehicle control (DMSO) and thrombin were used as negative and positive control respectively.

1.4 Confocal microscopy

Peptides were used to a final concentration of 10 µM. Confocal imaging of live washed platelet suspensions ($2x10^8$/mL) were conducted using a Ziess 510 confocal microscope with a Plan-APOCHROMAT 63x/1.4NA oil immersion objective, and a confocal aperture of 60 µm. Images of TAMRA-labelled model peptide stained platelets were acquired after 1 h incubation[1] using with excitation at 565 nm and emission was detected with a 560-615 nm BP filter. Images of FITC-peptide stained platelets

were acquired after 15 minutes incubation[1] using excitation at 488 nm and emission was detected with a 505-550 nm BP filter. Images showed that peptides **19**, **21** and SAHBa are able to enter the platelet cytosol. Peptide **17** showed poor cytosol-associated fluorescence while peptide **20** localized on the membrane and in the cytosol. Peptide **18** appeared to form fluorescent aggregates outside the platelets (Figure SI_5).

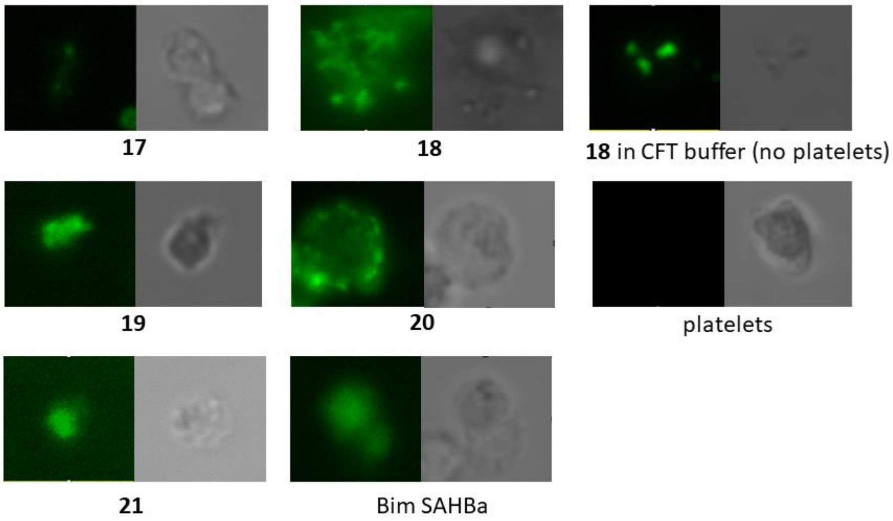

Figure SI_5. Images of platelets incubated with Bim-BH3 FITC-labelled peptides. For each peptide, the green channel is shown on the left and the gray on the right. Negative control (unstained platelets) and the ability of peptide **18** to form fluorescent aggregates in buffer are also shown.

1.5 Data analysis and statistics

Unless otherwise stated, the experiments were performed on no fewer than three separate occasions using platelets from different donors. Data were analysed using AccuriC6 BD Software and GraphPad Prism. Data are presented as mean values ± the standard error of the mean.

1.6 Circular dichroism

CD spectra were recorded on an Applied Photo-physics Chirascan circular dichroism spectro-polarimeter using a 1 mm path length quartz cuvette. CD measurements were performed at 298 K over a range of 185-250 nm using a response time of 0.5 s, 1 nm pitch and 0.5 nm bandwidth. The recorded spectra represent a smoothed average of three scans, zero-corrected at 250 nm and normalized (ϑ is quoted in

[1] Difference in incubation times are due to the fact that the model peptides do not activate platelets while the BH3 peptides do. Therefore, to avoid complication in the imaging due to platelet aggregation or activation images were acquired after 15 minutes incubation.

mdeg). Peptide **17** and **22** were dissolved in 1:1 ACN:MQ water to a final concentration of 50 µM. The spectra of peptides **19** and **20** were not recorded since the unstructured functionalised tag (4 to 11 amino acids) could interfere in the analysis.

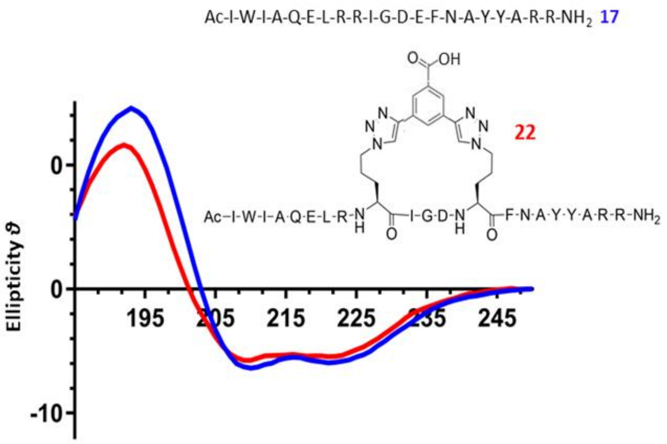

Figure SI_6. Circular dichroism of peptides **17** (blue) and **22** (red)

1.7 Serum stability test

One mL of RPMI-1640 media supplemented with 25% (v/v) of human serum was allocated into an Eppendorf tube and temperature kept at 37 °C for 15 minutes before commencing the experiment. 40 µL of the peptide in DMSO were added to make a final peptide concentration of 100 µg/mL. At specific intervals, 100 µL of the reaction mixture was taken and quenched with 200 µL of a 1:1 mixture of 96% Ethanol:DMSO. The suspension was cooled to 4 °C for 15 minutes and then spun at 13400 g for 4 minutes.[2] To 100 µL of the supernatant was then added caffeine (0.5 µL of 7 mg/mL solution in MQ water) as a reference. The supernatant was analyzed using C-18 HPLC with an eluting gradient 5-95% ACN (0.05% TFA) in water (0.05% TFA) over 15 minutes. Percentage of intact starting peptide was monitored over time (calculated as ratio of the area of the peak corresponding to the intact peptide to the area of the peak of caffeine). The results are the average of three independent repeats and the error is shown as SEM.

[2] Adapted from Serum Stability of peptides, Chapter 10, Peptide-based drug design, 2008, Springer Protocols

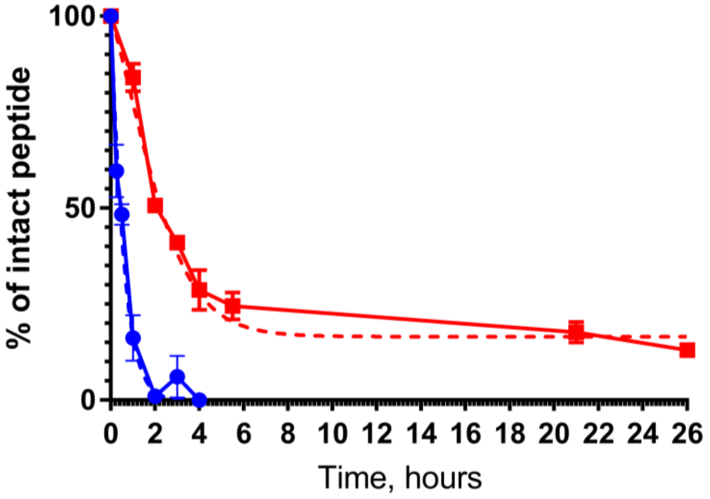

Figure SI_7. Peptide stability to human serum over 24 hours incubation at 37 °C. The linear peptide **17** is shown in blue and the stapled peptide **22** in red.

1.8 Protein expression and purification of His tagged BcL-xL

The His_6-TEV-Bcl-XL construct was expressed in *Escherichia coli* (BL21 Gold DE3) by induction with 0.2 mM IPTG for 16 h at 18 °C and purified by affinity purification followed by gel filtration. The pellets from 3 L culture were thawed and resuspended in lysis buffer (50 mM HEPES, pH 8.0, 500 mM NaCl, 20 mM imidazole, 5% glycerol, 1 mM TCEP) supplemented with protease inhibitors (Complete-EDTA Free, Roche Applied Science), Benzonase, and 2.5 mg/mL lysozyme. The lysate was clarified by centrifugation (15000 rpm, 60 min, 4 °C) and incubated with 8 mL of Talon Supeflow resin (Clontech) for 1 hour whilst rotating. The lysate was then passed through an Econocolumn (BioRad) and washed with 200 mL of lysis buffer. The protein was eluted by elution buffer (50 mM Hepes pH 8, 500 mM NaCl, 250 mM imidazole, 1 mM TCEP, 5% glycerol), and dialysed overnight against 5 L of dialysis buffer (50 mM Hepes pH 8, 300 mM NaCl, 1 mM TCEP). The dialysed protein was concentrated to 4 mL volume using centrifugal 10 k MWCO concentrator (Millipore). The concentrated protein was loaded on to HiLoad 16/60 Superdex 75 PG (GE Healthcare) pre-equilibrated in the storage buffer (25 mM Hepes pH 7.4, 150 mM NaCl, 1 mM TCEP). The fractions containing BcL-xL protein from this size exclusion chromatography step were analysed on SDS PAGE, pooled, snap frozen, and stored at -80 °C.

pNG020 N-His-TEV-EF-pET24a(+)-**Bcl-XL** (deletion of 45-85 and deletion of 209-233)

Length	187 aa
Molecular Weight	21681.79

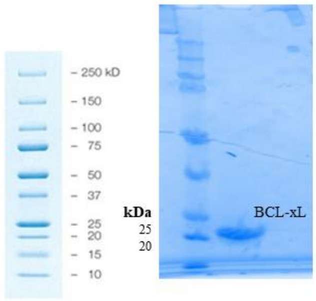

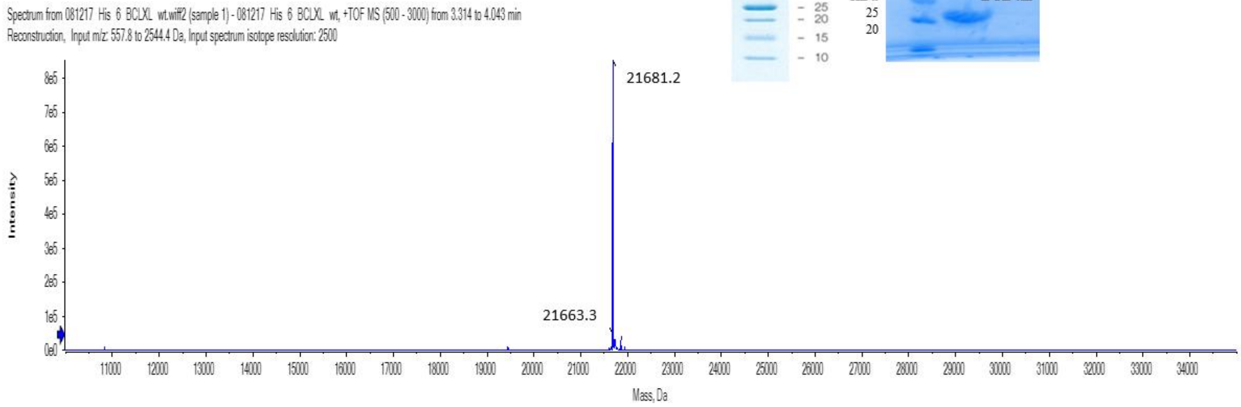

Figure SI_8. SDS gel of the purified protein, MS traces, length and MW of the Bcl-xL protein

1.9 Surface Plasmon Resonance (SPR)

The present method utilizes a Surface plasmon resonance (SPR)-based biosensor (Biacore^{TM,} GE Healthcare, Uppsala, Sweden) to characterize Bcl-xL peptide interaction partners.

Biacore^{TM} utilizes the phenomenon of surface plasmon resonance (SPR) to detect and measure binding interactions. In a typical Biacore experiment, one of the interacting molecules (ligand) is immobilized on a flexible dextran matrix while the interacting partner (analyte) is allowed to flow across that surface. A binding interaction results in an increase in mass on the sensor surface and a corresponding direct change in the refractive index of the medium in the vicinity of the sensor surface. Changes in refractive index or signal are recorded in resonance units (R.U.) Signal changes due to association and dissociation of complexes are monitored in a non-invasive manner, continuously and in real-time, the results of which are reported in the form of a sensorgram.

The SPR assay is configured to examine direct binding of peptides to Bcl-xL derivatized sensor surfaces and generate relative affinity values (K_{Dapp}) to provide relative ranking data for the series of peptides under examination.

Biacore^{TM} T200 (GE Healthcare, Uppsala, Sweden) was used to conduct all experiments reported herein. Sensor surface preparation and all interaction analyses experiments were performed at 25 ˚C utilizing a data collection rate of 10 Hz. Reagents were purchased from GE Healthcare. Running buffer containing 10 mM Hepes, pH 7.4,

150 mM sodium chloride, 1 mM dithiothreitol, 1% DMSO and 0.05% polysorbate 20 were utilized throughout surface preparation and all interaction analysis experiments.

Histidine tagged Bcl-xL protein was diluted to 5 µg/mL and captured onto Sensor chip NTA via the following sequence of injections to achieve a target Bcl-xL surface density of 700 R.U. (response units). The carboxymethyl dextran surface of Sensor chip NTA was activated by injection of an aqueous solution containing 50 mM N-hydroxy succinimide (NHS) and 200 mM N-ethyl-N'-(dimethylaminopropyl) carbodiimide (EDC). 500 µM nickel sulfate ($NiSO_4$) was subsequently injected for one minute and utilized to capture Bcl-xL from a 5 µg/mL solution injected for one minute. A further one-minute injection of 1M ethanolamine, pH 8.5 was performed to deactivate any unreacted carboxymethylesters.[3] The flow-rate for all injections during surface derivatization was 10 µl/min.

A blank flow-cell was similarly treated with the exception of injection of Bcl-xL and served as a reference surface in the direct binding interaction analyses.

Interaction analyses were performed by first equilibrating each sample within a 7-point 2-fold peptide dilution series in the range 33.3 nM to 1.03 nM for 30 minutes during instrument start-up procedures. nM peptide concentrations were used to accommodate potential solubility issues for the panel screening assay. Running buffer samples (0 nM peptide) were injected following each cycle and later used to facilitate double-referencing procedures during data analysis[4]. Each peptide sample was injected over each peptide surface 90 seconds at a flow-rate of 80 µL/min to monitor peptide association and peptide dissociation was subsequently monitored for 300 seconds. Data analyses were carried out using Biacore™ T200 evaluation software v2.0 to validate assay quality, perform double-referencing (blank buffer sample and reference surface data subtraction) and evaluate steady state affinity plots. Briefly, equilibrium R.U. levels (Req) were collected 5 seconds before the end of the sample injection. R.U. levels at this time-point were plotted versus sample concentration and the resultant binding isotherm fitted using the following steady state equation:

$$R_{eq} = Conc*R_{max}/(Conc+K_D)$$

R_{eq} refers to the equilibrium binding response (R.U.), R_{max} is the total surface binding capacity (R.U.) and K_D is the apparent equilibrium dissociation constant, a parameter subsequently utilized to provide a relative ranking for the peptides studied in the current panel.

---

[3] A. J. Kimple, R. E. Muller, D. P. Siderovski, F. S. Willard, *Methods in molecular biology (Clifton, NJ)*, 2010, **627**, 91-100.

[4] D. G. Myszka, *J. Mol. Recognit*. 1999, **12**(5), 279-84.

Table S1. Relative K_{dapp} of Bim BH3 stapled peptides

Peptide	Rel. K_{dapp} (M)	S.E. (K_{dapp})
**17**	7.27E-09	2.10E-10
**18**	>3.3E-08	
**19**	2.62E-08	2.80E-09
**21**	No binding	
Bim SAHBa	>3.3E-08	

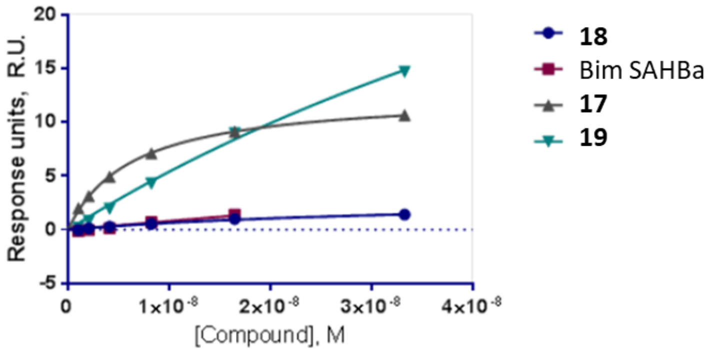

Figure SI_9. Overlay plot of steady state binding isotherms indicating peptides relative ranking.

1.10 General chemistry experimental procedure

The reagents were purchased from commercial sources and used without further purification. Dichloromethane was distilled from CaH_2.

Reactions were carried out under a dry nitrogen atmosphere at room temperature unless otherwise stated.

Thin layer chromatography was carried out on glass Merck Kieselgel 60 F254 plates, visualised by ultraviolet irradiation (254 and 365 nm) or by staining with ninhydrin (0.3% w/v in ethanol), prepared by standard procedures. Retention factors (R_f) are quoted to 0.01. Flash column chromatography was performed using Kieselgel 60 silica (230-400 mesh) with distilled solvents under a positive pressure of nitrogen.

^{1}H nuclear magnetic resonance spectra were recorded on Bruker Avance Ultrashield 400 or 500 spectrometers. Chemical shifts (δ) are quoted to the nearest 0.01 ppm and are referenced to the solvent residual peak. Coupling constants (*J*) are reported to the nearest 0.5 Hz. Data are reported as follows:

chemical shift, multiplicity (br, broad; s, singlet; d, doublet; t, triplet; q, quartet; m, multiplet; or a combination of these), coupling constant(s), integration and assignment.

^{13}C nuclear magnetic resonance spectra were recorded on Bruker Avance Ultrashield 400 or 500 spectrometers. Assignments are supported by DEPT-135, HSQC and HMBC spectra where necessary. Chemical shifts are quoted to the nearest 0.1 ppm and are referenced to the solvent residual peak.

Infra-red spectra were recorded neat on a Perkin Elmer Spectrum One FT-IR spectrophotometer fitted with an attenuated total reflectance (ATR) sampling accessory. Absorption maxima are reported in wavenumbers (cm^{-1}).

High resolution mass spectrometry (HRMS) was carried out using a Waters LCT Premier Time of Flight mass spectrometer or Micromass Quadrupole-Time of Flight mass spectrometer. Reported mass values are within the error limits of ±5 ppm.

Liquid chromatography-mass spectrometry (LCMS) was run on an Agilent 1200 series LC with an ESCi Multi-Mode Ionisation Waters ZQ spectrometer using MassLynx 4.1 software. LC system: solvent A: 10 mM ammonium acetate + 0.1% formic acid in H_2O; solvent B: 95% ACN + 5% H_2O + 0.05% formic acid; column: Supelcosil ABZ+PLUS column (33 mm × 4.6 mm, 3 µm); gradient: 0.0-0.7 min: 0% B, 0.7-4.2 min: 0-100% B, 4.2-7.7 min: 100% B, 7.7-8.5 min: 100-0% B; DAD spectrum: 190 nm-600 nm, interval 2.0 nm, peak width 0.200 min).

Analytical HPLC was run on an Agilent 1260 Infinity using a Supelcosil ABZ+PLUS column (150 mm × 4.6 mm, 3 µm) eluting with a linear gradient system (solvent A: 0.05% (v/v) TFA in H_2O, solvent B: 0.05% (v/v) TFA in ACN) over 15 minutes at a flow rate of 1 mL/min.

Semi-preparative HPLC was run on an Agilent 1260 Infinity using a Supelcosil ABZ+PLUS column (250 mm × 21.2 mm, 5 µm) eluting with a linear gradient system (solvent A: 0.1% (v/v) TFA in H_2O, solvent B: 0.05% (v/v) TFA in ACN) over 20 minutes at a flow rate of 20 mL/min. HPLC was monitored by UV absorbance at 220 and 254 nm, with TAMRA-labelled peptides also monitored at 550 nm. Retention times (R_t) are reported to the nearest 0.01 min.

Peptide concentration and composition were determined by amino acid analysis at the Peptide Nucleic Acid Chemistry Facility at the Department of Biochemistry, University of Cambridge.

1.11 Synthesis of linear peptides

All the peptides were synthesized on a solid-phase support using an Fmoc-protecting group strategy on an automated microwave peptide synthesizer (Liberty Blue, CEM) on Rink Amide MBHA LL resin (0.19-0.35 mmol/g, 100−200 mesh). Coupling was conducted using Fmoc-protected amino acids (5 equiv), HATU (5 equiv), DIPEA (10 equiv). Arginine was coupled using double couplings for 15 minutes each without microwave irradiation. All other amino acids were coupled using single couplings with 25 W power at 75 ^oC for 15 minutes with the exception of peptides **17**-**22** (and labelled analogues) for which all the amino acids were double coupled. Fmoc deprotection was carried out with 20% piperidine in DMF, using 45 W power at 75 ^oC over 3 min. The N-terminus of the peptide was capped by acetylation (peptides **12**, **17-22**) or coupled with TAMRA-Ahx-COOH (peptides **1**-**11**, **13-16**). The resin-bound peptides **17**-**21**-FITC were capped with Ahx and then coupled with 2 equiv of FITC and 4 equiv of DIPEA overnight. N-terminal capping with TAMRA was performed manually by treating the resin-bound peptide with TAMRA (6 equiv), HATU (6 equiv) and DIPEA (12 equiv) in DMF for 3 h. Peptides were cleaved from the resin in a TFA cocktail containing 92.5% (v/v) TFA, 2.5% (v/v) H_2O, 2.5% (v/v) triisopropylsilane, and 2.5% (v/v) dichloromethane either at rt for 3 h or at 42 °C for 30 minutes. The cleavage mixture was filtered to remove the resin, and the filtrate was dried. The cleaved peptides were precipitated with diethyl ether then purified via semi-preparative RP-HPLC. The pure peptides were lyophilized, the mass analyzed by LCMS and purity by analytical HPLC.

1.12 Synthesis of azido amino acids

Imidazole-1-sulfonyl azide hydrochloride (s1)

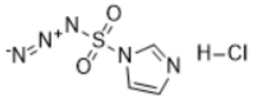

Sulfuryl chloride (16,1 mL), was added dropwise over 30 minutes to an ice-cooled suspension of NaN_3 (13.0 g, 200 mmol) in ACN (200 mL). The mixture was stirred at room temperature for 16 h. Imidazole (25.9 g, 380 mmol) was added to the ice-cooled and the resulting slurry stirred at rt for 5 h. The mixture was diluted with EtOAc (400 mL) and H_2O (400 mL). The organic layer was washed with H_2O (400 mL) and saturated $NaHCO_3$ (2 x 400 mL), dried over $MgSO_4$ and filtered. The volume was reduced *in vacuo* to 200 mL. Concentrated H_2SO_4 (11 mL) in EtOAc (100 mL) was added dropwise over 30

minutes at 0 °C and the resulting suspension was stirred at rt for 16 h. The precipitate was filtered, washed with EtOAc (3 x 60 mL) and dried *in vacuo* to give the title compound as white needles.

δ_H (400 MHz, d_6-DMSO): 8.64 (1H, s), 7.97 (1H, s), 7.35 (1H, s); **δ_C** (126 MHz, d_6-DMSO): 138.2, 130.9, 119.4

Data in accordance with literature.[5]

Fmoc-azido ornithine (s2)

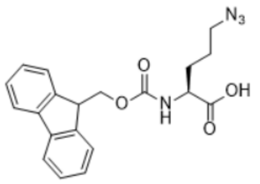

HCl in 1,4-dioxane (4 M, 14 mL) was added to a solution of Fmoc-Orn(Boc)-OH (2.00 g, 4.39 mmol) in 1,4-dioxane (14 mL). After stirring at rt for 16 h, the solvent was removed under a stream of nitrogen and the resulting residue was suspended in Et_2O (70 mL). The solid was filtered and washed with Et_2O (70 mL) to give the Fmoc-Orn-OH HCl as a white solid (1.70 g, 99%).

Fmoc-Orn-OH HCl (2.96 g, 7.54 mmol) was dissolved in a biphasic mixture of H_2O (44 mL), MeOH (87 mL) and CH_2Cl_2 (72 mL). $CuSO_4$ $5H_2O$ (14.5 mg, 0.058 mmol) and imidazole-1-sulfonyl azide hydrochloroide **s1** (5.80 g, 7.58 mmol) was added. The mixture was adjusted to pH 9 with saturated aqueous K_2CO_3 solution. After stirring vigourosly for 18 h, the organic solvents were removed *in vacuo*. The remaining aqueous phase was washed with Et_2O (2 x 50 mL), acidified to pH 2 with concentrated aqueous HCl and extracted with Et_2O (3 x 50 mL). The organic extracts were dried over $MgSO_4$ and concentrated *in vacuo*. The oily residue was dissolved in EtOAc and the solvent removed under a stream of nitrogen to give **s2** as a white solid (2.25 g, 5.91 mmol, 78%).

δ_H (400 MHz, d_6-DMSO): 7.89 (2H, d, *J* = 7.6 Hz), 7.74-7.68 (2H, m), 7.42 (2H, app t, *J* = 7.5 Hz), 7.33 (2H, app tt, *J* = 7.4, 1.1 Hz), 4.32-4.23 (4H, m), 3.39-3.32 (2H, m), 1.99-1.55 (4H, m, H4); **δ_C** (126 MHz, d_6-DMSO): 174.1, 156.6, 144.3, 141.2, 128.1, 127.5, 125.7, 120.6, 66.1, 53.8, 50.7, 47.1, 28.4, 25.5; $[\alpha]_D^{20}$ -2.02 (MeOH).

Data in accordance with literature.[6]

[5] N. Fischer, E. D. Goddard-Borger, R. Greiner, T. M. Klapötke, B. W. Skelton, J. Stierstorfer, J. Org. Chem. 2012, 77, 1760–1764.

[6] Y. H. Lau, P. De Andrade, S.-T. Quah, M. Rossmann, L. Laraia, N. Sköld, T. J. Sum, P. J. E. Rowling, T. L. Joseph, C. Verma, D. P. Lane, D. R. Spring, *Chem. Sci.*, 2014, **5**, 1804.

1.13 Synthesis of peptidic staples

Synthesis of the linkers of peptides **3**-**15**, **19-20** was achieved by manual solid-phase synthesis on Rink Amide resin using Fmoc-protecting group strategy and N-terminal capping with 3,5-diethynyl benzoic acid (2 equiv). HATU (2 to 4 equiv) was used as the coupling reagent and DIPEA (4 to 8 equiv) as the base.

Synthesis of 3,5-diethynyl benzoic acid

Methyl 3,5-bis((trimethylsylil)ethynyl)benzoate (s3)

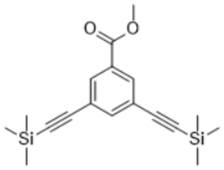

Trimethylsilylacetylene (6 mL, 42.2 mmol) was added to a stirring mixture of methyl 3,5-dibromobenzoate (900 mg, 3.10 mmol), $Pd_2(dba)_3$ (54 mg, 0.059 mmol), CuI (11.4 mg, 0.006 mmol) and PPh_3 (77.4 mg, 0.29 mmol) in dry triethylamine (15 mL). The reaction mixture was refluxed for 16 h under nitrogen. The solvent was removed *in vacuo*, the residue was diluted with EtOAc (30 mL) and washed with H_2O (3 x 30 mL). The organic phase was dried over $MgSO_4$ and the crude residue was purified via flash chromatography on silica gel eluting with a stepping gradient 0-5% EtOAc in Petrol Ether (PE) 40-60 to give **s3** as a yellow oil.

R_f = 0.4 (5% EtOAc:40-60 PE); **m.p.** 73-75 °C; **δ_H** (400 MHz, $CDCl_3$): 8.06 (2H, app d, *J* = 1.44 Hz), 7.74 (1H, app t, *J* = 1.32), 3.92 (3H, s), 0.26 (18H, s); **δ_C** (101 MHz, $CDCl_3$): 165.7, 139.0, 132.6, 130.5, 123.9, 102.9, 96.1, 52.4, 0.2

Data in accordance with literature.[3]

3,5-diethylbenzoic acid (s4)

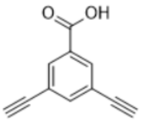

Aqueous 6 M KOH (3 mL, 17.9 mmol) was added to a stirred solution of **s3** (590 mg, 1.79 mmol) in MeOH (4 mL). The mixture was stirred at rt for 18 h. MeOH was removed under a stream of nitrogen and the aqueous phase was acidified to pH 4 with 6N HCl and extracted with EtOAc (3 x 5 mL). The combined organic phases were evaporated *in vacuo* to afford **s4** as orange solid (300 mg, 1.76 mmol, 98%).

R_f = 0.4 (20% MeOH/DCM); **δ_H** (400 MHz, MeOD): 8.05 (H, app d, *J* = 1.32), 7.72 (1H, app s), 3.66 (3H, s); **δ_C** (101 MHz, MeOD): 166.4, 138,5, 132.6, 131.6, 123.3, 81.0, 79.2

Data in accordance to literature.[7]

1.14 Synthesis of poly-guanidine biphenyl SMoC staple

2,3,2',3'-Tetra-{2-[*N*, *N*'-bis(tert-butoxycarbonyl)guanidino]-ethyloxy}-4-(aminomethyl)biphenyl prepared according to literature procedures.[8]

***N*-{2,3,2',3'-Tetra{2-[*N, N*'-bis(tert-butoxycarbonyl)guanidino]-ethyloxy}- biphenyl-4-ylmethyl}-3,5-diethynylbenzamide (s5)**

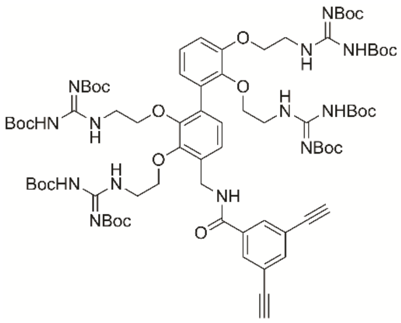

3,5-diethynylbenzoic acid (10.0 mg, 58.8 nmol) was added to a stirring solution of 2,3,2',3'-tetra{2-[*N, N'*-bis(tert-butoxycarbonyl)guanidino]-ethyloxy}-4-(aminomethyl)biphenyl (30 mg, 21.6 nmol), HATU (16 mg, 42.1 nmol) and DIPEA (10 uL, 57.5 mmol) in DMF (1 mL) at rt under nitrogen. The reaction mixture was stirred for 16 h. The solvent was removed *in vacuo*. The residue was purified by silica column chromatography eluting with ethyl acetate/*n*-hexane (1:1) to give the title compound as colorless oil (20 mg, 13.0 nmol, 60%).

δ_H (400 MHz, $CDCl_3$): 11.56 (s, 1H), 11.48 (s, 1H), 11.44 (s, 1H), 11.37 (s, 1H), 8.88 (s, 1H), 8.82 (s, 1H), 8.58 (s, 1H), 8.48 (s, 1H), 8.05 (s, 2H), 7.67 (s, 1H), 7.16 (d, *J* = 8.0 Hz, 1H), 7.05-7.00 (m, 2H), 6.92-6.89

[7] B. Förster, J. Bertran, F. Teixidor, C. Viňas, *J. Organomet. Chem*., 1999, **587**, 67-73
[8] D. S. AS Rebstock, C. Visintin, E. Leo, C. G. Posada, S. R. Kingsbury, G. H. Williams, K. Stoeber, *ChemBioChem* 2008, ***9***, 1787–1796.

(m, 2H), 4.61 (d, *J* = 5.0 Hz, 2H), 4.24-4.17 (m, 4H), 3.94-3.85 (m, 8H), 3.52-3.48 (m, 4H), 3.10 (s, 2H), 1.50-1.40 (m, 72H); **δ_C** (101 MHz, $CDCl_3$): 165.6, 163.3, 156.5, 156.3, 156.1, 156.0, 153.3, 153.1, 153.0, 151.8, 150.0, 149.1, 145.2, 137.8, 135.3, 132.5, 132.4, 131.6, 131.4, 126.8, 125.2, 124.1, 123.9, 123.0, 113.0, 83.5, 83.3, 83.1, 82.0, 79.5, 79.3, 78.7, 77.3, 71.9, 71.2, 70.6, 67.2, 53.5, 41.1, 40.2, 39.8, 28.4, 28.4, 28.3, 28.2, 28.1, 28.1, 28.0; **IR** (neat): 3335 (NH), 2979, 1714 (C=O), 1637, 1614, 1568, 1413, 1355, 1319, 1222, 1138, 1044, 1023, 808, 775; **HRMS** (m/z) for $C_{76}H_{109}N_{13}O_{21}{}^{23}Na_1$ $[M+H]^+$ calc. 1562.7753, found: 1562.7766.

***N*-[2,3,2',3'-Tetra(2-guanidino-ethyloxy)-biphenyl-4-ylmethyl]-3,5-diethynylbenzamide (s6)**

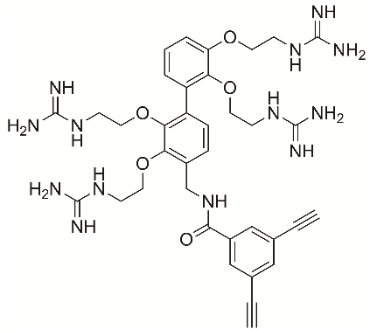

Trifluoroacetic acid/H_2O/triisopropylsilane 95/2.5/2.5 (2 mL) was added to a stirring solution of *N*-{2, 3, 2', 3'-Tetra{2-[*N, N*'-bis(tert-butoxycarbonyl)guanidino]-ethyloxy}- biphenyl-4-ylmethyl}-3,5-diethynylbenzamide **s5** (20 mg, 13.0 nmol) in dichloromethane (2 mL). The reaction mixture was stirred at rt for 3 h under nitrogen. The solvent was removed *in vacuo* to give the title compound as a colorless oil (9 mg, 12.2 nmol, 94%).

δ_H (500 MHz, MeOD): 7.99 (d, *J* = 1.5 Hz, 2H), 7.72 (s, 1H), 7.18-7.15 (m, 2H), 7.10 (d, *J* = 8.0 Hz, 1H), 7.04 (d, *J* = 8.0 Hz, 1H), 6.88 (d, *J* = 7.5 Hz, 1H), 4.67 (s, 2H), 4.29-4.23 (m, 2H), 4.22 (t, *J* = 5.0 Hz, 2H), 4.00-3.98 (m, 2H), 3.91 (t, *J* = 5.0 Hz, 2H), 3.70-3.66 (m, 6H), 3.32-3.28 (m, 2H), 3.25 (t, *J* = 5.0 Hz, 2H); **δ_C** (126 MHz, MeOD) δ: 168.2, 163.3, 163.0, 159.1, 159.0, 158.9, 152.8, 150.4, 150.3, 146.3, 139.0, 136.2, 134.0, 133.8, 133.6, 132.0, 127.8, 125.9, 124.9, 124.8, 119.3, 117.0, 114.8, 82.4, 80.9, 72.7, 72.6, 72.5, 68.1, 49.9, 49.6, 43.2, 43.0, 42.9, 42.3, 39.6; **IR** (neat): 3331 (br NH, NH_2), 3183, 2924, 1670 (C=O), 1459, 1431, 1354, 1320, 1265, 1202, 1138, 1048, 836, 801, 722 cm^{-1}; **HRMS** (m/z) for $C_{36}H_{46}N_{13}O$ $[M+H]^+$ calc. 740.3739, found: 740.3719.

1.15 Two-component double CuAAC peptide stapling

The double-click reaction was carried out to cross-link the azido-functionalised peptides with dialkynyl functionalised linkers.

All solvents for the reaction were degassed with nitrogen for 30 minutes before use. The dialkynyl linker (1.1 equiv) was added to the diazido-peptide in 1:1 (v/v) H_2O and tert-butanol under nitrogen before the addition of a solution of copper(II) sulfate pentahydrate (1 equiv), tris(3-hydroxypropyltriazolylmethyl)amine (THPTA, 1 equiv), and sodium ascorbate (3 equiv) in H_2O. The reaction mixture was stirred for 15 min-2 h at room temperature and monitored by LCMS. The crude product was purified on the semi-preparative HPLC as described above. The purified stapled peptides were lyophilized and analysed by LCMS and purity assessed by analytical HPLC. The absence of the azido groups in the product was monitored by IR (checking the absence of the $-N_3$ signal at around 2200 cm^{-1}).

2. Peptide mass-spectrometry characterization data

The mass of the peptidic staples and stapled peptides was characterized by LCMS.

Table S2. LCMS data of peptidic staples.

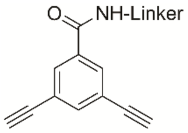

Linker	Mass	m/z found	m/z calculated	Species
s4- R-NH_2	325.2	326.1	326.1	$[M+H]^+$
s4- R2-NH_2	481.8	241.6	241.6	$[M+2H]^{2+}$
s4- R3-NH_2	637.4	319.6	319.7	$[M+2H]^{2+}$
s4-K-NH_2	297.2	298.3	298.2	$[M+H]^+$
s4-K2-NH_2	426.2	427.2	427.2	$[M+H]^+$
s4- K3-NH_2	554.3	553.3	553.2	$[M-H]^-$
s4- S3-NH_2	430.1	431.2	431.2	$[M+H]^+$
s4- E3-NH_2	556.2	555.3	555.2	$[M-H]^-$
s4- K3-OH	554.3	553.3	553.2	$[M-H]^-$
s4- (Ahx)-PKKRKV -NH_2	1146.7	1148.3	1147.7	$[M+H]^+$
s4- $(Ahx)_2$-PKKRKV -NH_2	1259.8	1261.3	1260.8	$[M+H]^+$
s4- $(Ahx)_3$-PKKRKV -NH_2	1372.9	1374.3	1373.9	$[M+H]^+$

Ahx = amino hexanoic acid spacer

Table S3. LCMS data of stapled peptides.

Peptide	Mass	m/z found	m/z calculated	Species
TAMRA Linear-p53	2239.1	1120.7	1120.6	$[M+2H]^{2+}$
**1**	2332.0	1166.4	1166.5	$[M+2H]^{2+}$
**2**	2418.3	1209.6	1209.6	$[M+2H]^{2+}$
**3**	2564.3	856.2	856.1	$[M+3H]^{3+}$
**4**	2720.4	908.3	908.5	$[M+3H]^{3+}$
**5**	2876.5	960.5	960.4	$[M+3H]^{3+}$
**6**	2536.4	1270.4	1270.2	$[M+2H]^{2+}$
**7**	2664.6	2665.3	2665.5	$[M+H]^{+}$
**8**	2792.4	1397.6	1397.7	$[M+H]^{+}$
**9**	2669.3	891.3	891.4	$[M+3H]^{3+}$
**10**	2795.3	1399.2	1399.2	$[M+2H]^{2+}$
**11**	2793.4	932.7	932.8	$[M+3H]^{3+}$
**12**	2081.0	1042.1	1042.1	$[M+2H]^{2+}$
**13**	3385.8	1130.0	1130.0	$[M+3H]^{3+}$
**14**	3498.9	1167.7	1167.7	$[M+3H]^{3+}$
**15**	3612.0	1205.3	1205.3	$[M+3H]^{3+}$
**16**	2978.5	994.1	994.2	$[M+3H]^{3+}$
**17**	2682.5	1341.6	1341.2	$[M+2H]^{2+}$
FITC-Ahx-**17**	3141.5	1629.9	1629.5	$[M+2H+TFA]^{2+}$
**18**	2975.2	1487.6	1487.6	$[M+2H]^{2+}$
FITC-Ahx-**18**	3216.5	1072.9	1073.5	$[M+3H]^{3+}$
**19**	3316.2	1658.1	1658.1	$[M+2H]^{2+}$
FITC-Ahx-**19**	3777.5	1889.3	1889.25	$[M+2H]^{2+}$
**20**	3940.0	1314.1	1313.3	$[M+3H]^{3+}$
FITC-Ahx-**20**	4399.0	1467.4	1467.0	$[M+3H]^{3+}$
**21**	3273.7	1638.4	1637.8	$[M+2H]^{2}$
FITC-Ahx-**21**	3733.4	1868.2	1868.2	$[M+2H]^{2+}$
**22**	2847.2	1424.9	1424.6	$[M+2H]^{2+}$
Bim SAHBa	2646.5	1324.2	1324.3	$[M+2H]^{2+}$
FITC-Ahx-Bim SAHBa	3109.6	1555.8	1555.8	$[M+2H]^{2+}$

3. ^{1}H-NMR and ^{13}C-NMR spectra

Imidazole-1-sulfonyl azide hydrochloride s1

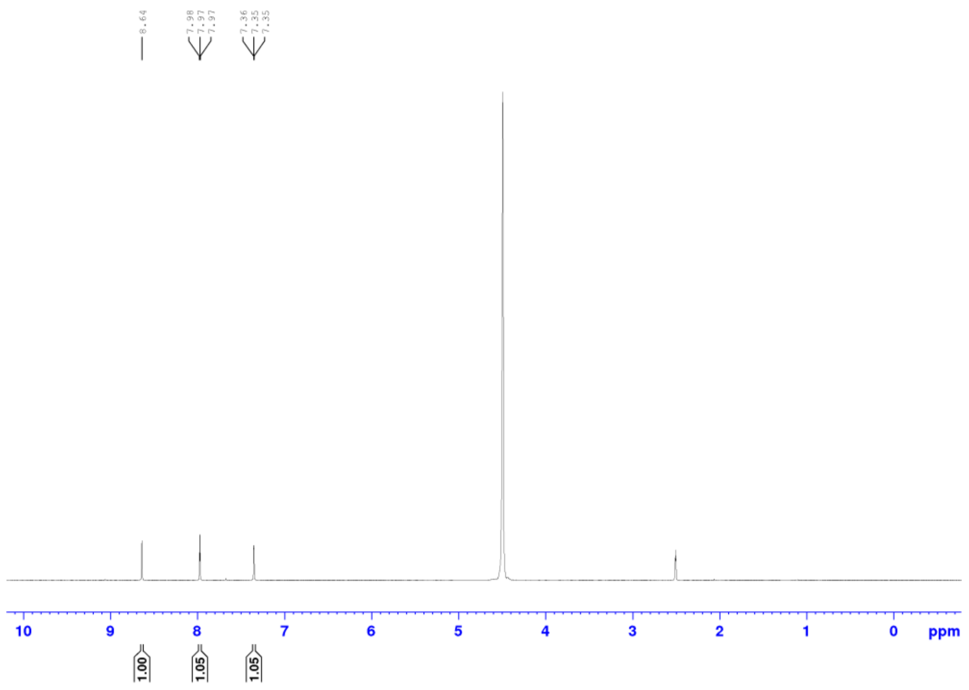

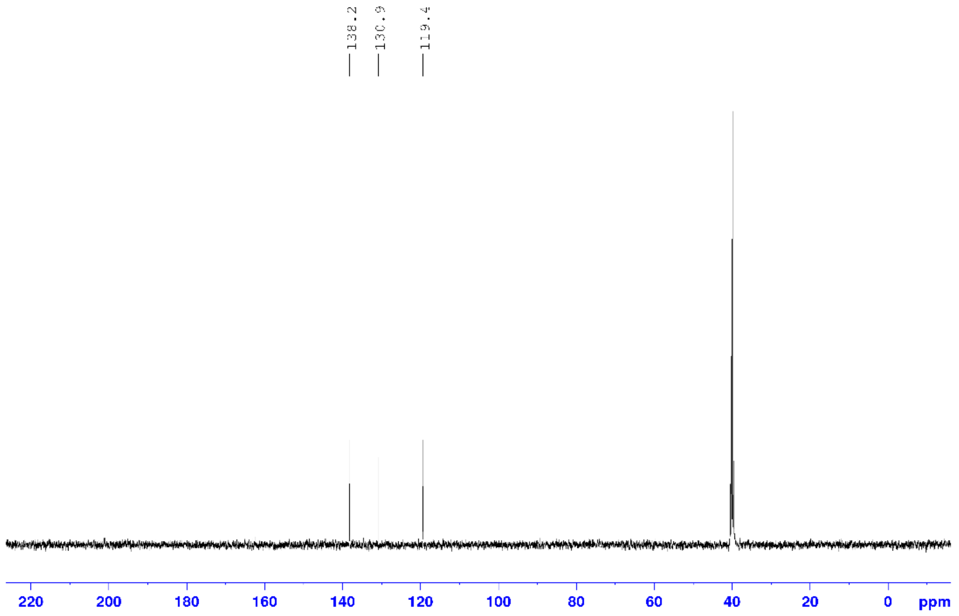

Fmoc-azido ornithine s2

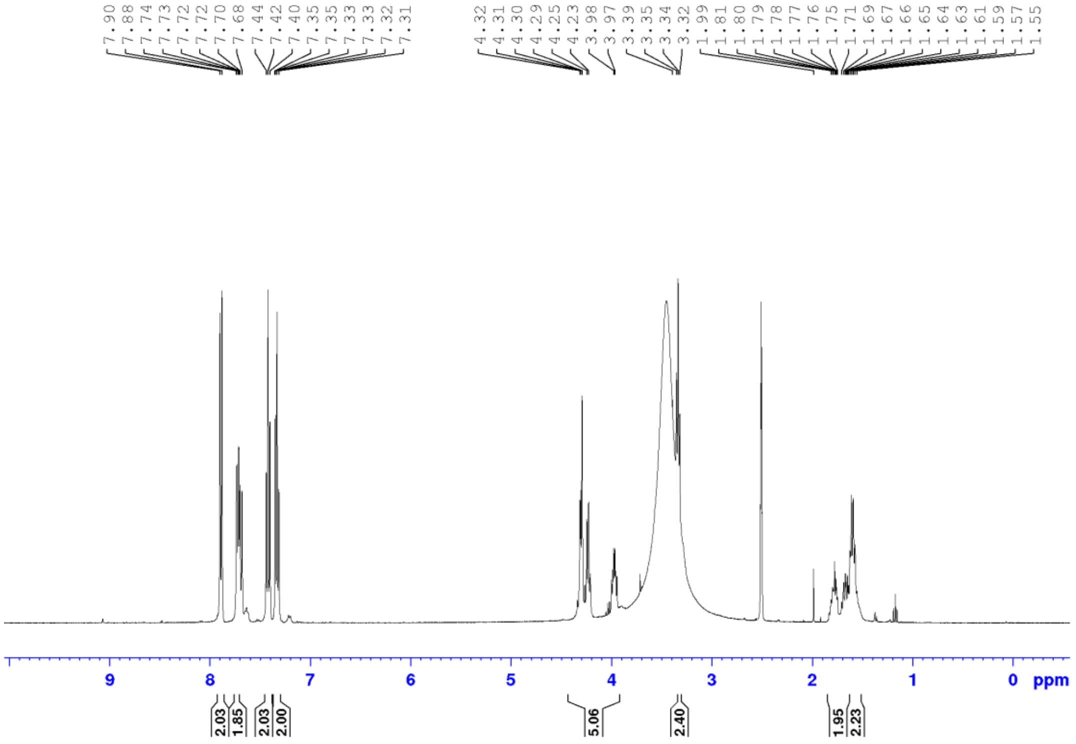

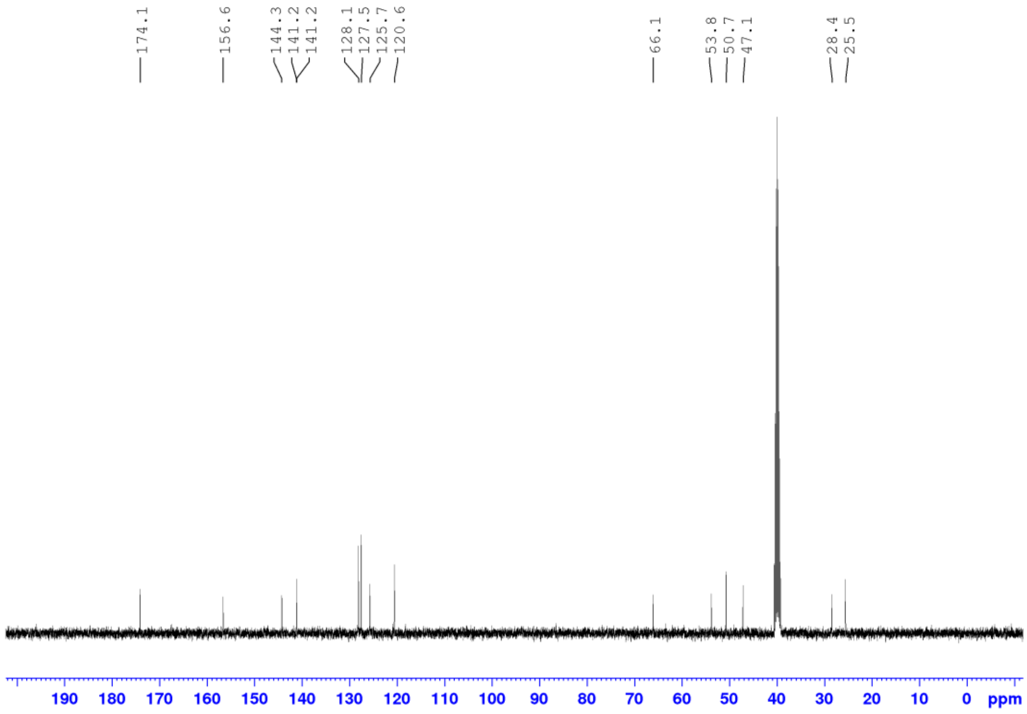

Methyl 3,5-bis((trimethylsylil)ethynyl)benzoate s3

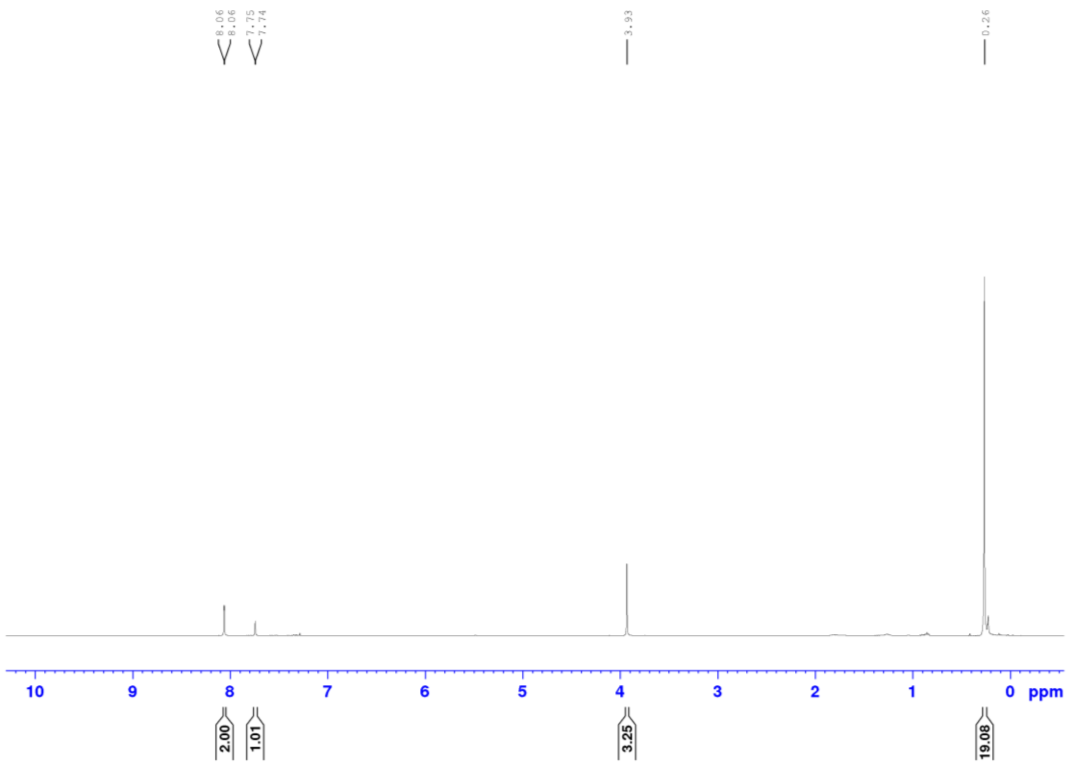

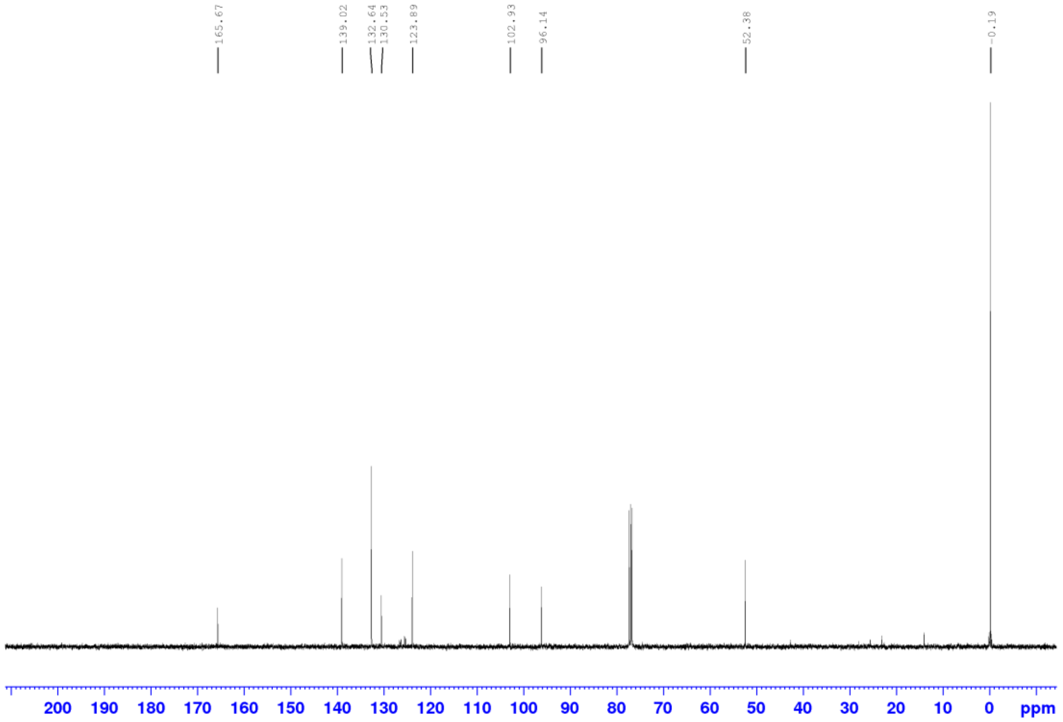

3,5-diethylbenzoic acid s4

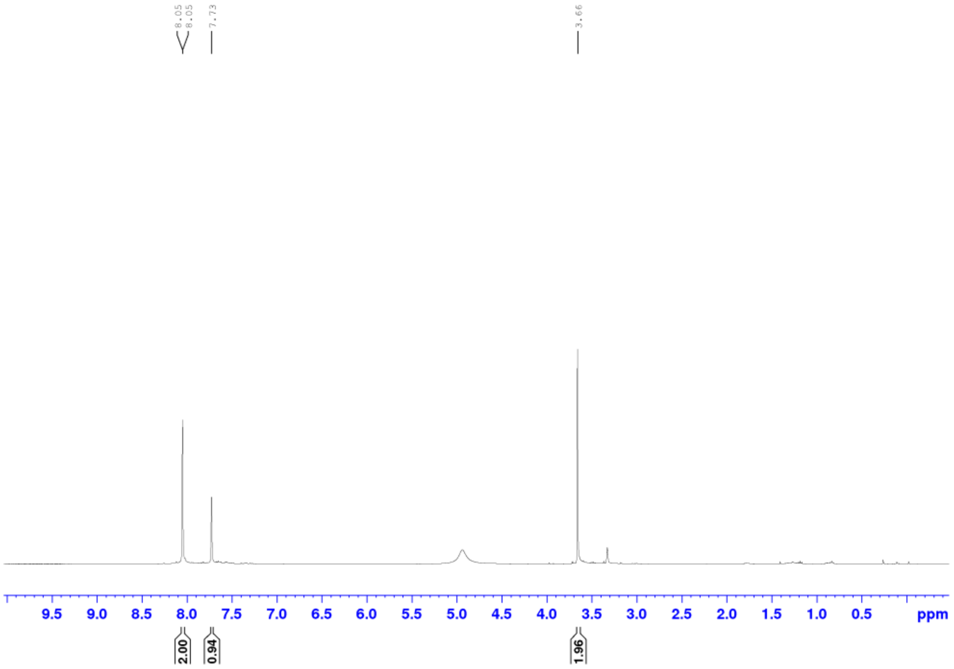

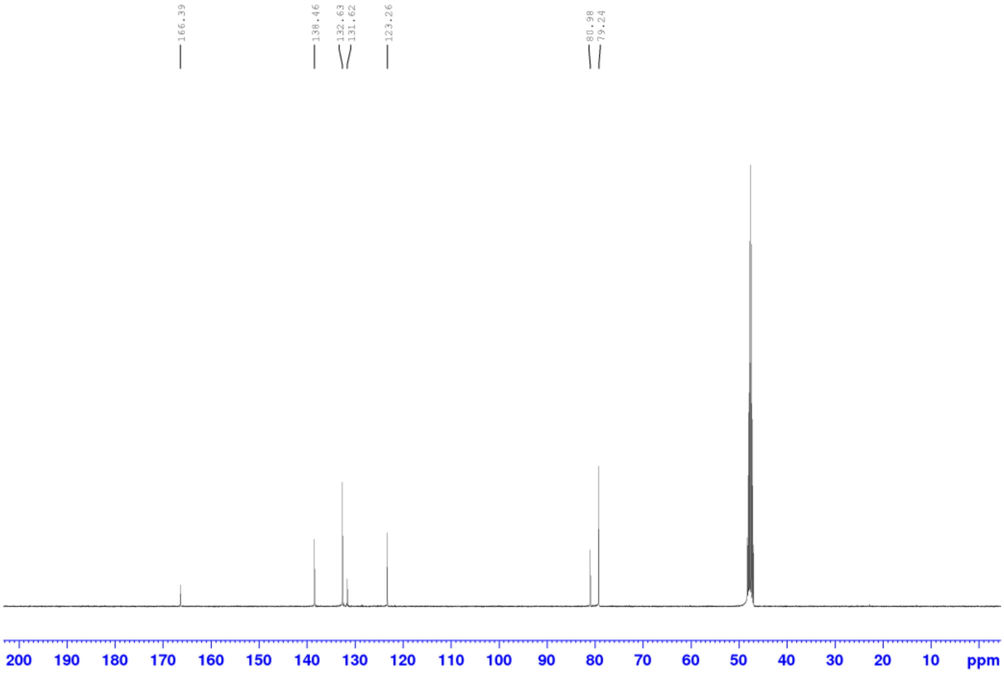

***N*-{2,3,2',3'-Tetra{2-[*N, N*'-bis(tert-butoxycarbonyl)guanidino]-ethyloxy}- biphenyl-4-ylmethyl}-3,5-diethynylbenzamide s5**

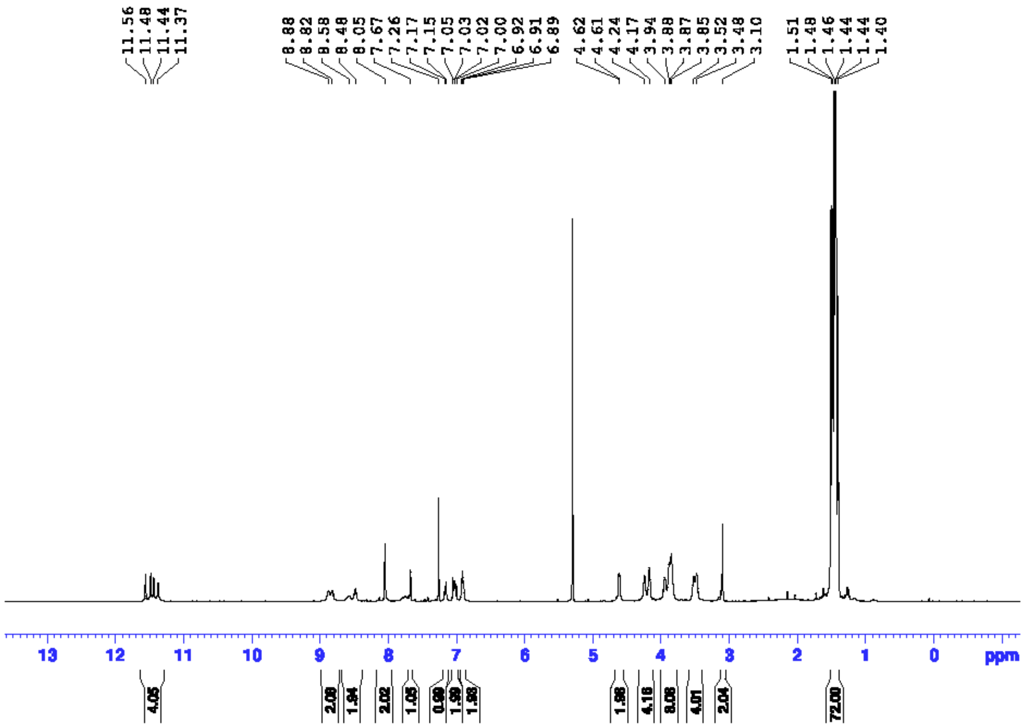

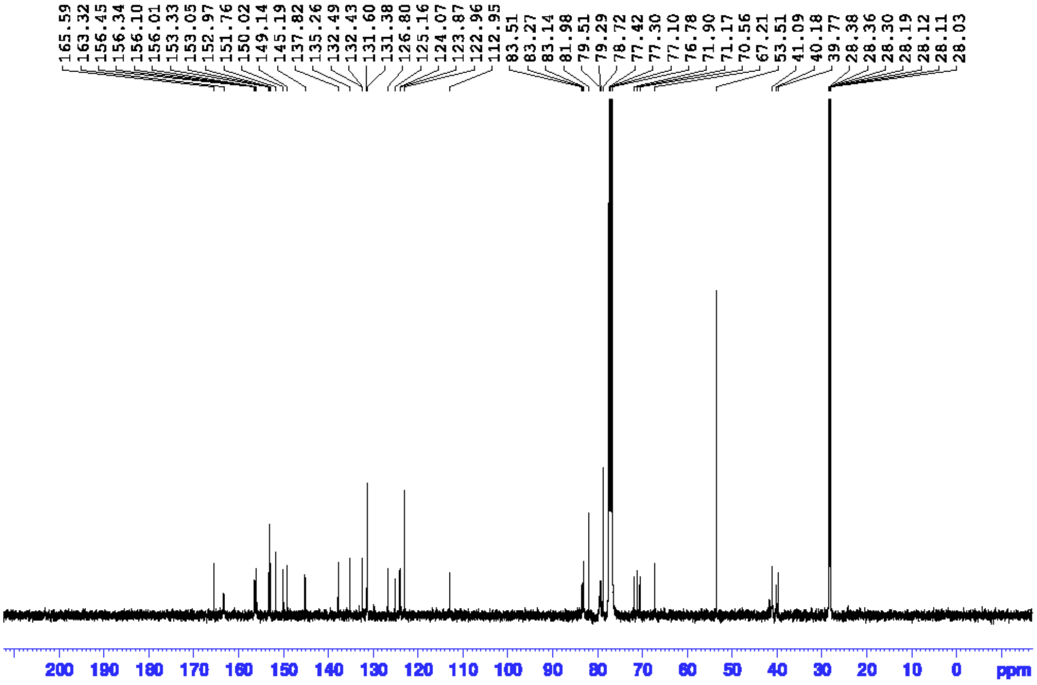

***N*-[2, 3, 2', 3'-Tetra(2-guanidino-ethyloxy)-biphenyl-4-ylmethyl]-3,5-diethynylbenzamide s6**

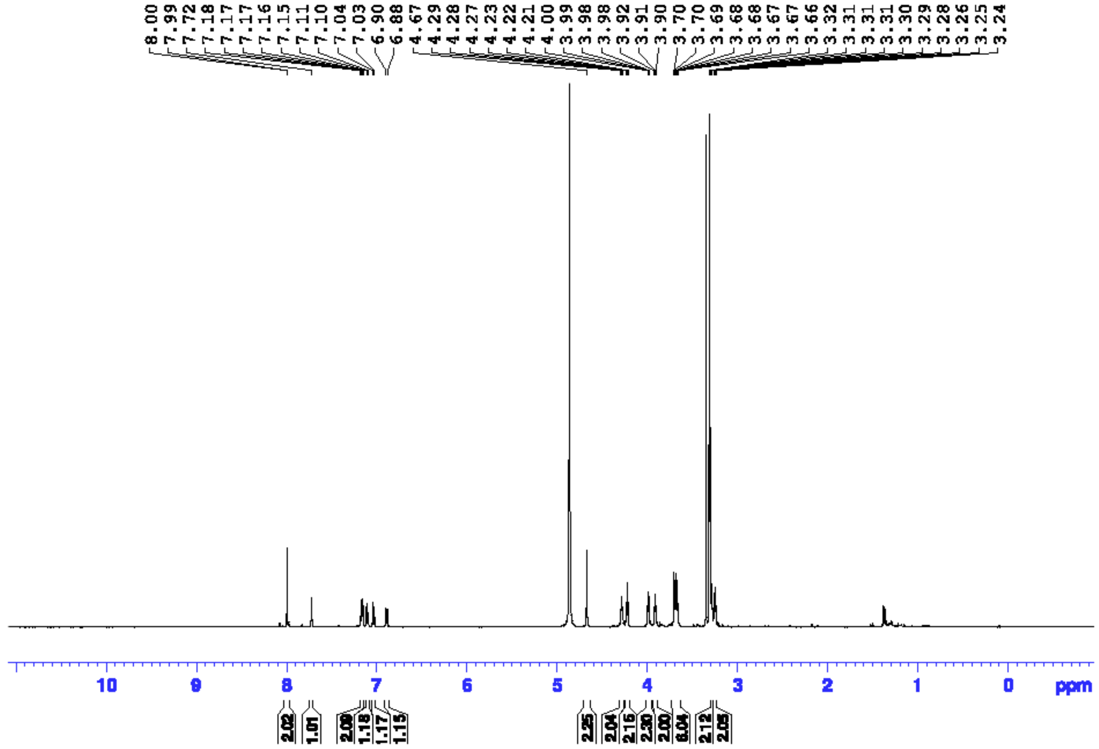

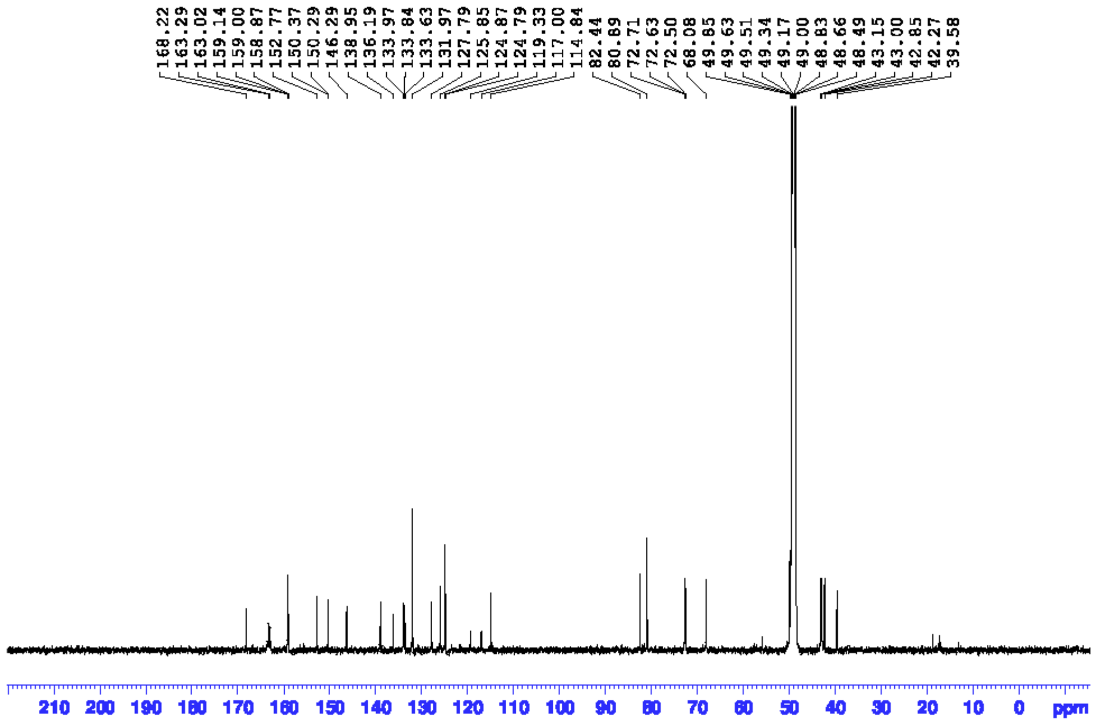

4. Molecular modelling

4.1 Preparation of structures

The structure of Bcl-xL (Δ45-84) in complex with Bim BH3 (residues 141-166) (PDB code 4QVF[9]) was used as the initial structure for molecular dynamics (MD) simulations. Bim BH3 was truncated to residues 146-166. The unresolved Bim residues 165 and 166 were then added using PyMOL to give peptide **17**. Bcl-xL was capped at its N- and C-termini by acetyl and N-methyl groups respectively while peptide **17** was capped at its N- and C-termini by acetyl and amide groups respectively. Peptide **17** was modified into the stapled peptide by replacing residues 154 and 158 with a two-component triazole staple formed by a double-click reaction between two azido-ornithine residues and 1,3-diethylnylbenzene. PDB 2PQR[10] was used to determine the protonation states of residues. Each Bcl-xL complex was solvated with TIP3P water molecules[11] in a periodic truncated octahedron box, such that its walls were at least 9 Å away from the complex, and neutralised with sodium ions.

4.2 Molecular dynamics

Energy minimisations and MD simulations were performed with the sander and PMEMD modules of AMBER 14[12] respectively. Three independent MD simulations were carried out on each of the Bcl-xL complexes using the ff14SB[13] and generalized AMBER force fields (GAFF).[14] Atomic charges for the stapled residues were derived using the R.E.D. Server,[15] which fits restrained electrostatic potential (RESP) charges[16] to a molecular electrostatic potential (MEP) computed by the Gaussian 09 program[17] at the HF/6-31G* theory level. All bonds involving hydrogen atoms were constrained by the SHAKE

---

[9] S. Rajan, M. Choi, K. Baek and H. S. Yoon, *Proteins: Struct. Funct. Bioinform.*, 2015, **83**, 1262-1272.

[10] T. J. Dolinsky, P. Czodrowski, H. Li, J. E. Nielsen, J. H. Jensen, G. Klebe and N. A. Baker, *Nucleic Acids Res.*, 2007, **35**, W522-W525

[11] W. L. Jorgensen, J. Chandrasekhar, J. D. Madura, R. W. Impey and M. L. Klein, *J. Chem. Phys.*, 1983, **79**, 926-935.

[12] D. A. Case, V. Babin, J. T. Berryman, R. M. Betz, Q. Cai, D. S. Cerutti, T. E. Cheatham, III, T. A. Darden, R. E. Duke, H. Gohlke, A. W. Goetz, S. Gusarov, N. Homeyer, P. Janowski, J. Kaus, I. Kolossváry, A. Kovalenko, T. S. Lee, S. LeGrand, T. Luchko, R. Luo, B. Madej, K. M. Merz, F. Paesani, D. R. Roe, A. Roitberg, C. Sagui, R. Salomon-Ferrer, G. Seabra, C. L. Simmerling, W. Smith, J. Swails, R. C. Walker, J. Wang, R. M. Wolf, X. Wu and P. A. Kollman, *AMBER* 2014.

[13] J. A. Maier, C. Martinez, K. Kasavajhala, L. Wickstrom, K. E. Hauser and C. Simmerling, *J. Chem. Theory Comput.*, 2015, **11**, 3696-3713.

[14] J. M. Wang, R. M. Wolf, J. W. Caldwell, P. A. Kollman and D. A. Case, *J. Comput. Chem.*, 2004, **25**, 1157-1174.

[15] E. Vanquelef, S. Simon, G. Marquant, E. Garcia, G. Klimerak, J. C. Delepine, P. Cieplak and F.-Y. Dupradeau, *Nucleic Acids Res.*, 2011, **39**, W511-W517.

[16] W. D. Cornell, P. Cieplak, C. I. Bayly and P. A. Kollman, *J. Am. Chem. Soc.*, 1993, **115**, 9620-9631.

[17] M. J. Frisch, G. W. Trucks, H. B. Schlegel, G. E. Scuseria, M. A. Robb, J. R. Cheeseman, G. Scalmani, V. Barone, B. Mennucci, G. A. Petersson, H. Nakatsuji, M. Caricato, X. Li, H. P. Hratchian, A. F. Izmaylov, J. Bloino, G. Zheng, J. L. Sonnenberg, M. Hada, M. Ehara, K. Toyota, R. Fukuda, J. Hasegawa, M. Ishida, T. Nakajima, Y. Honda, O. Kitao, H. Nakai, T. Vreven, J. Montgomery, J. A., J. E. Peralta, F. Ogliaro, M. Bearpark, J. J. Heyd, E. Brothers, K. N. Kudin, V. N. Staroverov, R. Kobayashi, J. Normand, K. Raghavachari, A. Rendell, J. C. Burant, S. S. Iyengar, J. Tomasi, M. Cossi, N. Rega, N. J. Millam, M. Klene, J. E. Knox, J. B. Cross, V. Bakken, C. Adamo, J. Jaramillo, R. Gomperts, R. E. Stratmann, O. Yazyev, A. J. Austin, R. Cammi, C. Pomelli, J. W. Ochterski, R. L. Martin, K. Morokuma, V. G. Zakrzewski, G. A. Voth, P. Salvador, J. J. Dannenberg, S. Dapprich, A. D. Daniels, Ö. Farkas, J. B. Foresman, J. V. Ortiz, J. Cioslowski and D. J. Fox, *Gaussian 09, Revision B.1*, Gaussian, Inc., Wallingford CT, 2009.

algorithm,[18] allowing for a time step of 2 fs. Nonbonded interactions were truncated at 9 Å, while the particle mesh Ewald method[19] was used to account for long range electrostatic interactions under periodic boundary conditions. Weak harmonic positional restraints with a force constant of 2.0 kcal mol^{-1} $Å^{-2}$ were placed on the protein and peptide non-hydrogen atoms during the minimisation and equilibration steps. Energy minimisation was carried out using the steepest descent algorithm for 500 steps, followed by the conjugate gradient algorithm for another 500 steps. The systems were then heated gradually to 300 K over 50 ps at constant volume before equilibration at a constant pressure of 1 atm for another 50 ps. Subsequent unrestrained equilibration (2 ns) and production (100 ns) runs were carried out at 300 K using a Langevin thermostat[20] with a collision frequency of 2 ps^{-1}, and 1 atm using a Berendsen barostat[21] with a pressure relaxation time of 2 ps. A snapshot of peptide **22** in complex with Bcl-xL at the end of the simulation is shown in Figure SI_10.

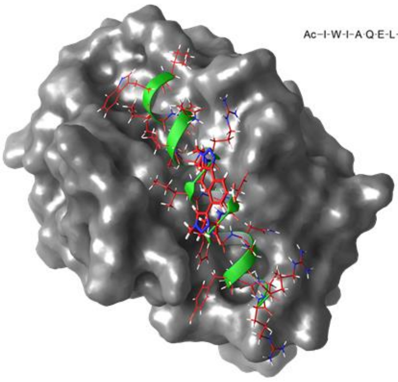

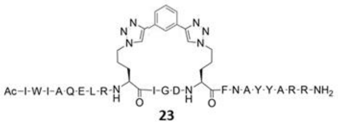

22

Figure SI_10. Peptide **22** in complex with Bcl-xL at the end of the MD simulation. The protein surface is shown in grey, the secondary structure of the peptide in green, residues in red sticks and the staple as red tubes.

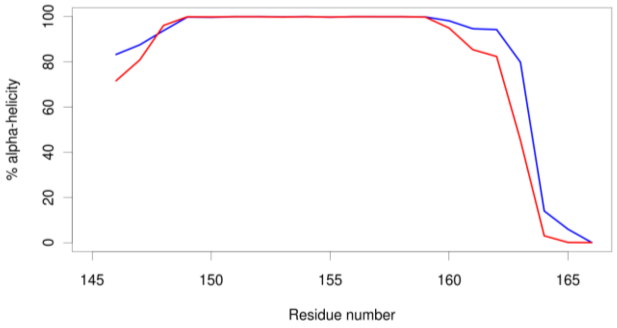

Figure SI_11. Average percentage α-helicity of residues in peptides **17** (blue) and **22** (red) when they are bound to Bcl-xL during the MD simulations.

[18] J. P. Ryckaert, G. Ciccotti and H. J. C. Berendsen, *J. Comput. Phys.*, 1977, **23**, 327-341.

[19] T. Darden, D. York and L. Pedersen, *J. Chem. Phys.*, 1993, **98**, 10089-10092.

[20] J. A. Izaguirre, D. P. Catarello, J. M. Wozniak and R. D. Skeel, *J. Chem. Phys.*, 2001, **114**, 2090-2098.

[21] H. J. C. Berendsen, J. P. M. Postma, W. F. Vangunsteren, A. Dinola and J. R. Haak, *J. Chem. Phys.*, 1984, **81**, 3684-3690.

4.3 Binding free energy calculations

Binding free energies for the Bcl-xL complexes were calculated using the molecular mechanics/generalized Born surface area (MM/GBSA) method[22] implemented in AMBER 14.[8] Two hundred equally-spaced snapshot structures were extracted from the last 40 ns of each of the trajectories, and their molecular mechanical energies calculated with the sander module. The polar contribution to the solvation free energy was calculated by the pbsa[23] program using the modified generalised Born (GB) model described by Onufriev *et al.*[24] while the nonpolar contribution was estimated from the solvent accessible surface area using the molsurf[25] program with γ = 0.0072 kcal Å^{-2} and β set to zero. Entropies were estimated by normal mode analysis[26] using the nmode program. Due to its computational expense, only 50 equally-spaced snapshots from the last 40 ns of the trajectories were used for entropy calculations. The results are shown in Table SI3.

Table SI4. Computed binding energies of linear and stapled peptides

Peptide	sequence	ΔH (kcal/mol)	TΔS (kcal/mol)	ΔG (kcal/mol)
**17**	Ac-IWIAQELRRIGDEFNAYYARR-NH_2	-108.29 ± 1.46	-52.59 ± 0.41	-55.70 ± 1.24
**22**	Ac-IWIAQELRXIGDXFNAYYARR-NH_2	-107.58 ± 4.94	-52.54 ± 1.91	-55.05 ± 3.07

[22] J. Srinivasan, T. E. Cheatham, P. Cieplak, P. A. Kollman and D. A. Case, *J. Am. Chem. Soc.*, 1998, **120**, 9401-9409.

[23] R. Luo, L. David and M. K. Gilson, *J. Comput. Chem.*, 2002, **23**, 1244-1253.

[24] A. Onufriev, D. Bashford and D. A. Case, *Proteins: Struct. Funct. Bioinform.*, 2004, **55**, 383-394.

[25] M. L. Connolly, *J. Appl. Crystallogr.*, 1983, **16**, 548-558.

[26] B. R. Brooks, D. Janezic and M. Karplus, *J. Comput. Chem.*, 1995, **16**, 1522-1542.
